# The DNA replication checkpoint targets the kinetochore to reposition DNA structure-induced replication damage to the nuclear periphery

**DOI:** 10.1016/j.celrep.2025.116083

**Published:** 2025-07-30

**Authors:** Tyler M. Maclay, Jenna M. Whalen, Matthew J. Johnson, Catherine H. Freudenreich

**Affiliations:** 1 Department of Biology, Tufts University, Medford, MA 02155, USA; 2 These authors contributed equally; 3 Lead contact

## Abstract

Hairpin-forming CAG/CTG repeats pose significant challenges to DNA replication. In *S. cerevisiae*, long CAG/CTG repeat tracts reposition from the interior of the nucleus to the nuclear pore complex (NPC) to maintain their integrity. We show that relocation of a (CAG/CTG)_130_ tract to the NPC is dependent on phosphorylation of Mrc1 (hClaspin) of the fork protection complex and activation of the Mrc1/Rad53 replication checkpoint, implicating an uncoupled fork as the initial damage signal. Dun1-mediated phosphorylation of the kinetochore protein Cep3 is required for repositioning, a constraint that can be overcome by centromere inactivation, connecting detachment of the kinetochore from microtubule ends to NPC association. Activation of this pathway leads to the formation of DNA damage-induced microtubules, which associate with the repeat and are necessary for locus repositioning. These data implicate the replication checkpoint in facilitating the movement of DNA structure-associated damage to the nuclear periphery by centromere release and microtubule-directed motion.

## In brief

Expanded CAG repeats, the cause of Huntington’s disease, stall replication and reposition to the nuclear periphery to effect repair. Maclay et al. report that the DNA replication checkpoint targets the kinetochore protein Cep3 for phosphorylation, which results in centromere release and repeat locus association with damage-inducible microtubules to promote relocation.

## Graphical Abstract

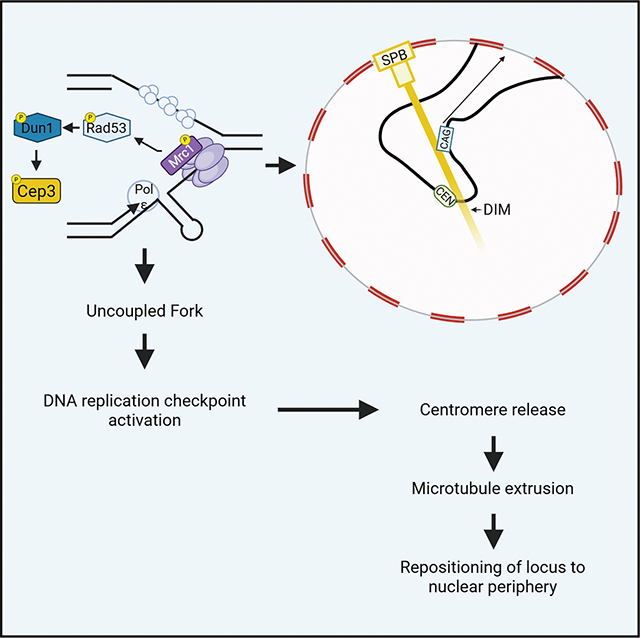

## INTRODUCTION

The nuclear periphery (NP) has emerged as an important site for the maintenance of genome stability. Several types of replication stress have been shown to provoke relocation to the NP in yeast and human cells. These include drugs known to stall or collapse replication forks, such as hydroxyurea (HU) combined with methyl methanesulfonate (MMS),^1^ expanded CAG repeats that form secondary structures,^2^ the polymerase inhibitor aphidicolin,^3^ protein-mediated stalls by replication fork barriers,^4,5^ and replication stalls within telomeres.^6,7^ Some persistent types of DNA damage such as double-strand breaks (DSBs) lacking a readily available template for repair or in sequestered heterochromatin domains and eroded telomeres also relocate the NP (for review, see the studies by Whalen and Freudenreich, Lamm et al., and Gasser and Stutz^8–10^).

We previously elucidated a pathway for how an expanded CAG/CTG tract of 130 repeat units (abbreviated (CAG)_130_) integrated into a *S. cerevisiae* chromosome relocates from the nuclear interior to the nuclear pore complex (NPC) in late S phase in a manner dependent on replication.^2,11^ CAG/CTG tracts over 35 repeat units can form hairpin structures that can impair replication and increase chromosome breakage.^12,13^ Defects in relocation to the NPC result in increased chromosome end loss and repeat instability, implicating this pathway in protecting against fork breakage and inaccurate repair.^2^ The DNA structure-induced relocation pathway has many similarities with pathways that have been described for persistent or heterochromatic DSBs and eroded telomeres but also have several differences (reviewed in the study by Whalen and Freudenreich^8^). We determined that NPC association of the (CAG)_130_ repeat was dependent on resection, the binding and SUMOylation of key fork-associated repair proteins (RPA, Rad52, Rad59, Smc5) by the Smc5–6 associated SUMO ligase Mms21/Nse2, and interaction with SUMO-interacting motifs of Slx5, which targets the CAG locus to the NPC.^11^ However, it is unclear what type of replication-induced damage initiates this pathway. Possibilities include an uncoupled fork, a resected reversed fork, a broken fork, or post-replicative DNA damage left behind after fork restart. Forks that no longer have an intact replisome or break are often collectively referred to as collapsed forks, but recent results indicate that there are multiple types of fork collapse and restart depending on the location and type of damage.^14,15^

In addition to the SUMOylation requirements, we wanted to investigate other processes that could be involved in the relocation of replication damage caused by CAG repeats to the NPC. One important pathway known to respond to collapsed forks is the DNA damage response (DDR). The DDR can be subdivided into two pathways: the DNA replication checkpoint (DRC) and the DNA damage checkpoint (DDC), defined by their mediator proteins Mrc1 and Rad9, respectively. We previously showed that yeast cells containing expanded CAG repeats activate the DDC, which is evident by repeat-specific cell-cycle arrest phenotypes in wild-type cells and by Rad53 phosphorylation in repair-defective cells,^16^ and the long range resection required for (CAG)_130_ relocation^11^ would produce single-stranded DNA (ssDNA) to stimulate DDC activation. In addition, members of the DDC and DRC were important for maintaining repeat stability and preventing chromosome breaks at long CAG tracts.^17–19^

The checkpoint is composed of several sensor and mediator components that activate downstream effectors, reviewed in Hustedt et al., 2013^20^ and McClure et al., 2022.^21^ Sensor proteins identify processed DNA lesions and recruit other proteins to the damage site. In *S. cerevisiae*, the sensor Ddc2 (hATRIP) interacts with RPA-bound ssDNA to recruit the kinase Mec1 (hATR). Mec1 is also activated by the PCNA-like 9–1-1 clamp (Ddc1, Rad17, Mec3 in budding yeast), which is loaded by the Rad24-RFC complex onto double-stranded (ds)/ssDNA junctions. Downstream effector proteins such as Rad53 (hChk2) and Chk1 elicit the DDR through further phosphorylation signaling. At the replication fork, there is an additional Rad53 activation mechanism involving the mediator Mrc1 (hClaspin), which travels with the replisome and is phosphorylated upon replisome uncoupling.^22,23^ Another pathway involves Tel1 (hATM), which senses DNA ends through its interaction with the yeast MRX (hMRN) complex. Finally, the yeast mediator protein Rad9 (h53BP1) amplifies Rad53 phosphorylation. Rad9 is recruited to histone modifications, including methylated H3 (H3-K79) and damage-specific H2A-S129 phosphorylation, which can occur at both collapsed forks and DSBs. Due to the different DNA damage sensors, identification of the checkpoint pathway that is activated can give insights into the lesion or lesions that initiate the DDR.

Relocation of persistent DSBs in *S. cerevisiae* and heterochromatic DSBs in *Drosophila* required both Mec1/Tel1 and ATR/ATM, respectively.^1,24,25^ In human cells that were treated with aphidicolin to inhibit polymerases, ATR inhibition reduced the localization of PCNA foci to the NP.^3^ In contrast, single deletion of either Mec1 or Tel1 did not prevent the relocation of a (CAG)_130_ repeat to the NPC,^2^ leaving it an open question whether the checkpoint is involved in the nuclear repositioning of damage caused by DNA structures.

Here, we show that activation of the effector kinase Rad53 by phosphorylation of the replisome-associated Mrc1 protein is needed for the relocation of a (CAG)_130_ tract to the NP, implicating an uncoupled fork as the principal signal to initiate this pathway. We identified the Rad53 target Dun1 as an important factor for relocation. By investigating several Dun1 substrates, we determined that the phosphorylation of the kinetochore protein Cep3 at serine 575 is the critical target of Dun1 to allow for CAG tract repositioning. We investigated possible mechanisms by which Cep3 phosphorylation could act and identified the disruption of centromere attachment to the spindle pole body (SPB) (the yeast microtubule-organizing center, which is functionally equivalent to the mammalian centrosome) as a key factor. In contrast, Cep3 phosphorylation-dependent increases in global chromosome movement were not detectable. Finally, we show that damage-induced microtubules (DIMs)^26^ are formed in response to damage at the CAG tract, colocalize with the CAG tract locus, and are dependent on this checkpoint pathway. We conclude that the phosphorylation of Cep3-S575 allows for relocation of the damage induced by the structureforming repeat tract to the NPC by modulating centromere attachment and supporting DIM-directed repositioning toward the NP, defining an additional role for the replication checkpoint.

## RESULTS

### Relocation of a (CAG)_130_ tract to the NPC depends on the activation of Rad53 and Mrc1

**Figure 1.**
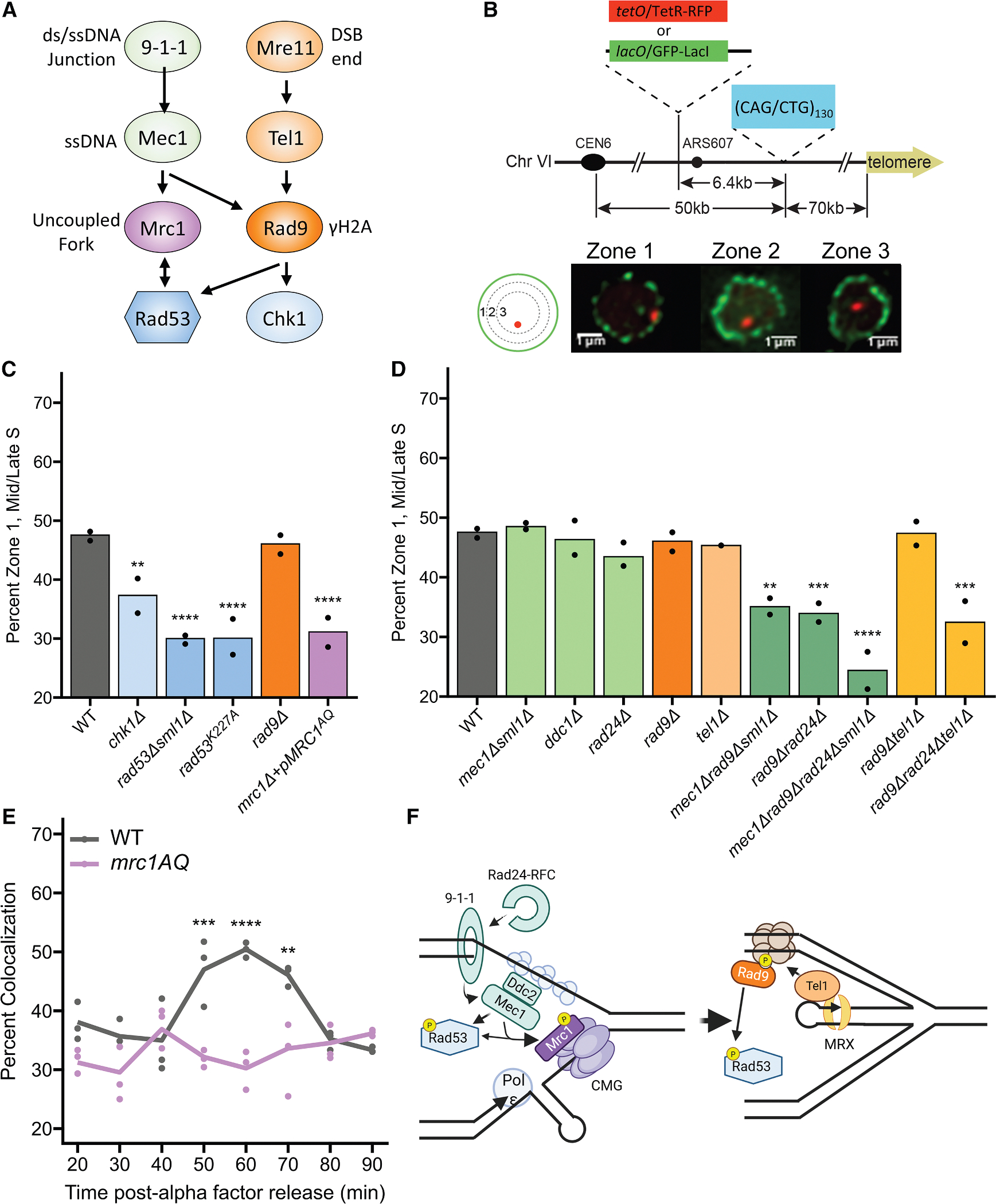
Relocation of a (CAG)_130_ locus to the NP depends on the activation of the DDC or DRC (A) Flowchart of the DNA damage checkpoint pathway. (B) Yeast chromosome 6 containing an integrated (CAG/CTG)_130_ repeat tract ((CAG)_130_) and a *lacO* or *tetO* array that binds the GFP-LacI or TetR-RFP protein, respectively. Mid-to-late S-phase cells were imaged, and the location of the foci was scored into one of three zones of equal area, using the GFP-Nup49 signal to mark the NP. Representative images are shown. Scale bars, 1 μm. (C and D) Percentage of cells with LacO loci in zone 1 for mid/late S phase cells for the indicated strains. Points represent the results of at least 2 biologically independent experiments done with independent isolates for mutants, and >120 cells (avg. ~200) were scored per genotype. See method details and Table S1 for the exact number of cells analyzed per experiment and genotype, percentages, and *p* values. Compared with the wild-type (CAG)_130_ strain by chi-squared test. (E) Percentage colocalization of CFP-LacI foci (representing the (CAG)_130_ locus) with the NP in wild-type (WT) or *mrc1*^*AQ*^ cells post alpha factor release. Points represent 3 biologically independent experiments, and >220 cells (avg. ~300) were scored per time point and genotype. Significance calculated using Bonferroni-corrected chi-squared test comparing wild type to mutant for each time point. Detailed in Table S2. *p ≤ 0.05, **p ≤ 0.01, ***p ≤ 0.001, ****p ≤ 0.0001. (F) Model illustrating the activation of the DRC in response to an uncoupled fork (left) or activation of the DDC following fork reversal and recruitment of the MRX complex (right). Created with Biorender.com.

We used a previously described zoning assay^2^ to visualize the CAG repeat in relation to the NP. Briefly, (CAG)_130_ was integrated on chromosome 6, 6.4 kb away from a LacO or TetO array, which can be visualized by binding of GFP-LacI or mCherry-TetR, respectively (Figure 1B). Fluorescently tagged Nup49 was used to mark NPCs to allow for visualization of the NP. The nucleus was divided into three equal areas to determine the frequency of CAG repeat occupancy in each zone (see STAR Methods). Zone 1 represents the NP (where the NPCs are located), zone 3 is the middle of the nucleus, and zone 2 is the area in between (Figure 1B). The presence of the (CAG)_130_ tract caused an increase in the occupancy of the nearby tagged locus in zone 1, from 31% in the (CAG)_0_ control to 48% for the chromosome containing (CAG)_130_ (Figure 1C). The distribution between zones 1, 2, and 3 was consistent between both wild-type (CAG)_130_ strains, whether the LacO/GFP-LacI or TetO/mCherry-TetR array was used to follow the CAG repeat locus (Figure S1A).

Previously, it was shown that single deletions in Mec1 or Tel1 sensor kinases do not affect relocation of the (CAG)_130_ tract to the NP during S phase.^2^ Simultaneous disruption of Mec1 and Tel1 could not be tested by the zoning assay due to the severely abnormal nuclear morphology. As there are many factors involved in the activation of the DDR that can act redundantly (Figure 1A), we investigated if there was any role for the checkpoint in CAG tract relocation by testing the effector kinases. Deletion of Chk1 showed a mild, yet significant, decrease in the frequency with which the CAG tract was found at the NP (Figure 1C). However, deletion of Rad53 (viable with the added deletion of Sml1^27^) resulted in a significant decrease in (CAG)_130_ occupancy in zone 1. This was further confirmed by testing *rad53*^*K227A*^, a kinase-defective mutant of Rad53 that is checkpoint deficient.^28^ *rad53*^*K227A*^ showed the same significant decrease in zone 1 occupancy as *rad53Δsml1Δ* (Figure 1C). We conclude that activation of the DDR and the kinase activity of Rad53 are needed for relocation of the expanded CAG tract locus to the NPC.

There are multiple sensor and mediator proteins that can activate Rad53 in response to different types of DNA damage. We had previously shown that long CAG tracts were especially prone to breakage and exhibited increased fork stalling in the absence of the Mrc1 protein,^19^ which couples polymerase epsilon to the CMG helicase. Mrc1 contains multiple SQ/TQ motifs that are targeted by the serine/threonine protein kinases of the PIKK family, such as Mec1, Tel1, or Rad53 for phosphorylation upon fork uncoupling.^29,30^ The Mrc1^AQ^ mutant, with its 17 SQ/TQ sites mutated to alanine,^30^ had a modest effect on chromosome fragility but exhibited increased repeat instability,^19^ suggesting a possible role for Mrc1 in sensing forks stalled at the (CAG)_130_ tract. The deletion of Mrc1 led to severely abnormal nuclear morphology, precluding zoning analysis. However, complementation with the checkpoint-deficient Mrc1^AQ^ mutant abrogated these abnormal morphologies and showed a significant reduction in the association of the repeat locus with the NP to a similar level as a Rad53 mutant (Figure 1C). Thus, the checkpoint signal relevant for repositioning to the NPC is channeled through Mrc1, which suggests that the signal originates from an uncoupled replication fork.

### Mec1 and Rad9 act redundantly to sense damage at the (CAG)_130_ tract and signal repositioning within the nucleus

Though single mutations in either of the main checkpoint sensor kinases, Mec1 and Tel1, did not result in a decrease in relocation to the NP (see Figure 1D), the presence of the Mre11 protein, which senses and processes DNA ends, is required.^11^ Deletion of the Rad9 mediator protein (h53BP1 ortholog), which facilitates Rad53 autophosphorylation,^31,32^ showed no effect on repeat positioning (Figure 1D). Similarly, deletion of the 9–1-1 clamp component Ddc1 (9–1-1 activates Mec1) or the clamp loader Rad24 also had no effect on the percent of the CAG locus in zone 1 (Figure 1D). These results were somewhat surprising given the important role of Mrc1, which is phosphorylated by Mec1, and our previous findings that both Mec1 and Rad9 were important for preventing breaks and instability at expanded CAG tracts.^17^ Since there is a well-described redundancy between these sensing pathways (reviewed in the study by Hustedt et al.^20^), we explored whether there was redundancy in the sensing of lesions to signal CAG tract repositioning. Deletion of both Mec1 and Rad9 resulted in a decrease in percent zone 1 occupancy of the (CAG)_130_ tract (Figure 1D). In addition, co-disruption of Rad9 and Rad24 led to a decrease in association with the NP, and deletion of all 3 genes (*mec1Δrad24Δrad9Δ*) led to the lowest percent zone 1 occupancy (Figure 1D). These effects were not made worse by the additional disruption of Tel1 (Figure 1D), suggesting that Tel1 could act in the same pathway as Rad9 in this context. We previously showed that both H2A-S129 and H2A-T126 phosphorylation could be detected at a (CAG)_155_ tract 40 min into S phase,^33,34^ which is shortly before relocation occurs^11^ and could serve to recruit Rad9.

Because the DDC has been shown to be activated later than the DRC,^35^ we sought to examine whether the relocation of the repeat was simply delayed in *mrc1*^*AQ*^ cells rather than absent. Examining colocalization of the repeat locus with the NP in *mrc1*^*AQ*^ cells revealed that the relocation defect does not resolve at later time points (Figure 1E). It was previously shown that Mrc1can be phosphorylated in response to HU in the absence of either Mec1 or Rad53, though at a reduced level, and phosphorylation in response to MMS damage was dependent on Rad9 and Rad53 but not Mec1.^36^ Therefore, Mrc1 could be phosphorylated by either the Mec1 kinase or the Rad9-activated Rad53 kinase at a CAG-hairpin stalled fork at a level sufficient to initiate a response, consistent with our results that Mec1 and Rad9 act redundantly with respect to the CAG tract-NPC repositioning pathway (Figure 1F).

### Phosphorylation of Cep3 serine 575 by Dun1 is needed for relocation of the CAG tract to the NPC

**Figure 2.**
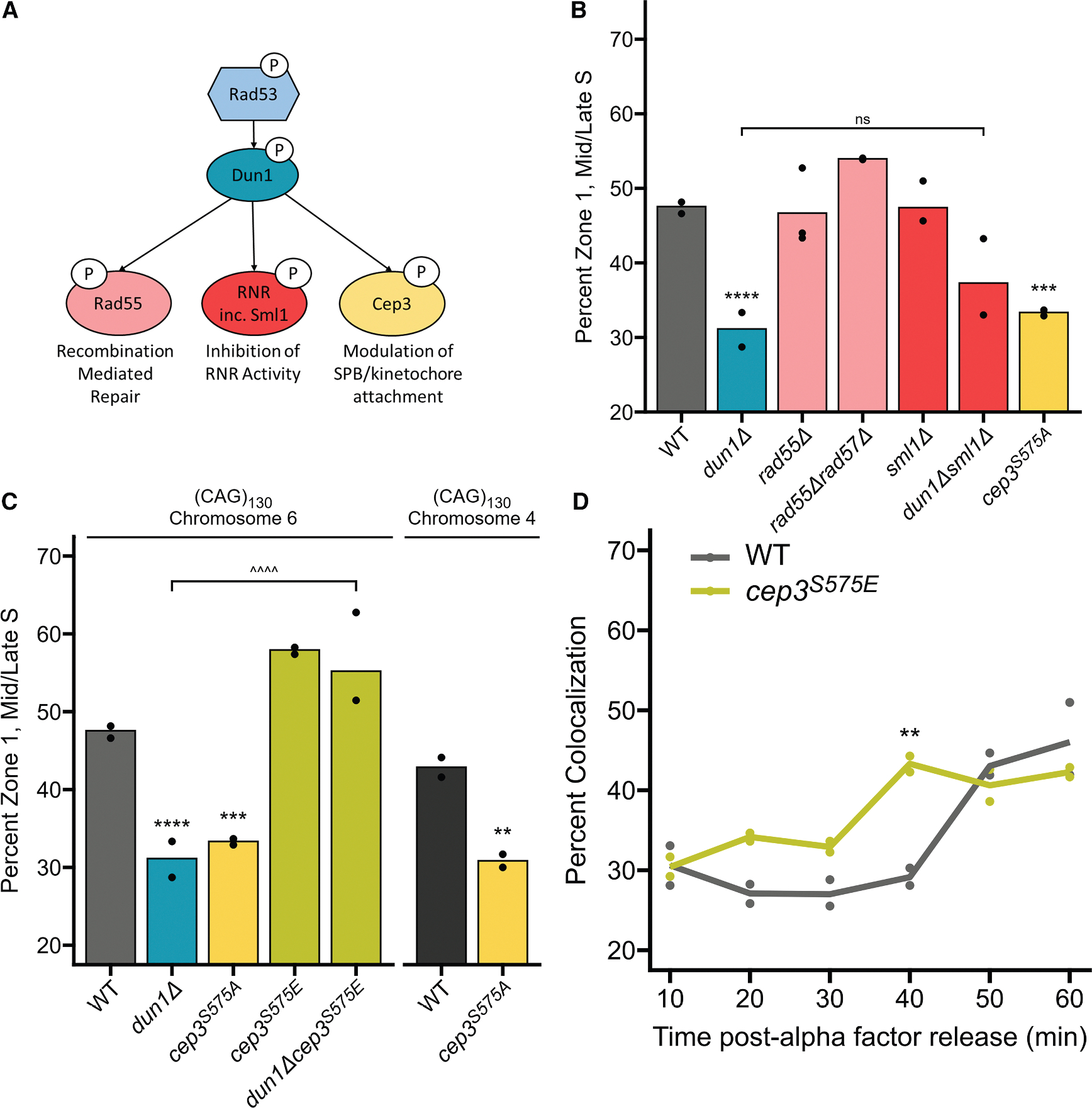
Phosphorylation of Cep3^S575^ by Dun1 is needed for relocation of (CAG)_130_ to the NP (A) Diagram of Dun1 targets. (B) Zoning analysis for strains deleted for Dun1 or its targets. (C) Zoning analysis for a phosphomimetic mutant of Cep3 (*cep3*^*S575E*^) individually and in the *dun1Δ* mutant. Chromosomal location as annotated. (^^^^) *p* ≤ 0.0001 compared as indicated by chi-squared test. Zoning performed and analyzed as described in Figure 1 (Table S1). (D) Percentage colocalization of the repeat locus and the NP in 2 biologically independent experiments of (CAG)_130_ cells at indicated time points after release from alpha factor arrest in wild-type and *cep3*^*S575E*^ strains. >200 cells (avg. 241) per time point were scored for each genotype (Table S3). Significance calculated by Bonferroni corrected chi-squared test. See also Figure S1C; Table S4 for equivalent zoning data. *p* values. *p ≤ 0.05, **p ≤ 0.01, ***p ≤ 0.001, ****p ≤ 0.0001 compared with the wild-type (CAG)_130_ strain by chi-squared test.

After determining that activation of the transducing kinase Rad53 by the DRC is required for (CAG)_130_ relocation, we investigated what targets of Rad53 are needed. Dun1 is a well-characterized target of Rad53 (Figure 2A).^37–40^ A *dun1Δ* strain showed a significant decrease in CAG tract association with the NPC similar to the *rad53Δsml1Δ* mutants (Figure 2B). Therefore, Rad53 activation of Dun1 is a crucial step in the pathway leading to repositioning of replication-induced damage to the NPC.

Next, we investigated what targets of Dun1 are required for relocation (Figure 2A). Dun1 is required for the phosphorylation of the repair protein Rad55,^37^ which allows dimerization with Rad57 and stabilizes Rad51 binding to promote successful strand invasion.^41^ However, neither *rad55Δ* nor *rad55Δrad57Δ* mutants showed a decrease in the percentage of CAG tract loci detected in zone 1 (Figure 2B). Dun1 has been well studied as an activator of ribonucleotide reductase (RNR) activity and functions to regulate nucleotide metabolism in response to replicative stress. Dun1 inhibits Rfx1, Sml1, and Dif1 through phosphorylation, which prevents them from downregulating nucleotide pools.^42–44^ Since Dun1 inhibits Sml1 activity, to determine if Dun1’s role in relocation is through maintaining nucleotide pools, Sml1 was deleted in the *dun1Δ* strain. If the deletion of Sml1 rescued the defect in CAG tract repositioning in the *dun1Δ* strain, it would indicate that this activity of Dun1 is required. However, *dun1Δsml1Δ* showed a similar percent S-phase occupancy in zone 1 as *dun1Δ* strains (Figure 2B). We conclude that the role for Dun1 in relocation to the NP is neither to promote recombination-mediated repair nor to increase nucleotide pools.

Another phosphorylation target of Dun1 is the kinetochore protein Cep3.^45^ Cep3 is a DNA-binding protein that recognizes the centromere sequence as a member of the CBF3 kinetochore complex.^46,47^ The yeast kinetochore interacts with the end of the microtubule emanating from the SPB, the yeast microtubule-organizing center that is functionally equivalent to the mammalian centrosome. Phosphorylation of Cep3 at serine 575 (S575) occurs in response to an induced DSB or zeocin-induced DNA damage in a manner dependent on both Rad53 and Dun1.^45^ Mutation of serine 575 to a non-phosphorylatable alanine residue resulted in a significant decrease in the CAG locus occupancy in zone 1, showing that Cep3 phosphorylation is required for repositioning of the CAG tract to the NPC (Figure 2B).

To confirm that Cep3 phosphorylation at S575 is required for repositioning, we constructed a phosphomimetic mutant of Cep3 by mutating serine 575 to glutamic acid. The *cep3*^*S575E*^ mutation bypassed the need for Dun1 as the *dun1Δcep3*^*S575E*^ mutant showed a normal level of CAG locus S-phase occupancy in zone 1 (Figure 2C). We conclude that Dun1-mediated phosphorylation of Cep3-S575 is needed for the relocation of damage resulting from collapsed forks to the NPC.

To further address the role of Cep3 phosphorylation, we assessed the timing of CAG locus relocation to the NP in *cep3*^*S575E*^ cells over the course of S phase. As previously reported, in wild-type cells, the (CAG)_130_ tract is rarely at the NPC in early S phase and is maximal in late S phase at 60 min post release from G1 (Figure 2D).^11^ However, Rad52 is found at the repeat locus 40 min into S phase,^2^ suggesting that fork collapse has occurred by then and that there is a delay between the occurrence of damage and anchoring at the pore. The *cep3*^*S575E*^ mutation led to a significant increase in the occupancy of the (CAG)_130_ repeat tract at the NP at the 40-min time point (Figure 2D). This suggests that the *cep3*^*S575E*^ phosphomimetic primes the fork for repositioning once repair proteins are recruited to the damaged locus.

### Cep3-S575 phosphorylation allows for CAG tract repositioning by modulating kinetochore-SPB attachment

**Figure 3.**
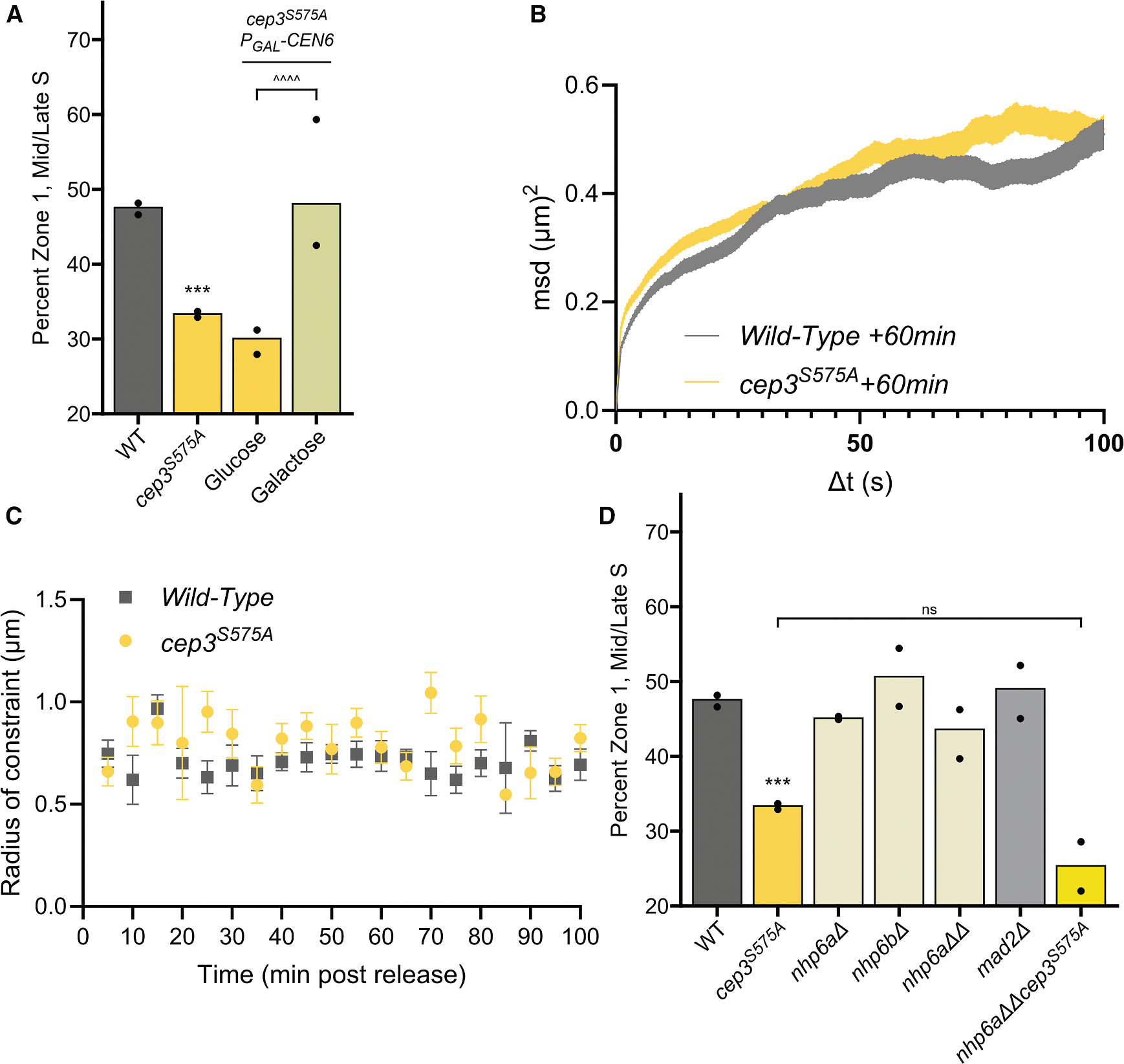
Cep3 phosphorylation allows for (CAG)_130_ relocation through release from the centromeric constraint (A) Zoning analysis for *cep3*^*S575A*^ and *P*_*GAL1*_*-CEN6 cep3*^*S575A*^ strains. Cultures were grown in YP-lactate media, then split into media containing either glucose or galactose to induce transcription through CEN6. (B) Example MSD track for *cep3*^*S575A*^ and wild-type strains containing (CAG)_130_. Data were captured 60 min post release from alpha factor arrest. Cells were imaged every 1.5 s for 5 min, first Δt = [0.100] s shown. The lines represent the 95% confidence intervals of the mean square displacement at a particular time point. (C) Summary of the radii of constraint calculated for wild-type and *cep3*^*S575A*^ strains over 100 min post release from alpha factor arrest. Points represent the calculated radius of constraint for a 5-min interval imaged every 1.5 s as in (B). Error bars are standard error of the calculated radius of constraint (Table S5). (D) Zoning analysis for strains defective for mobility or the SAC. Zoning performed and analyzed as described in Figure 1 (Table S1). *p* values. *p ≤ 0.05, **p ≤ 0.01, ***p ≤ 0.001, ****p ≤ 0.0001 compared with the wild-type (CAG)_130_ strain by chi-squared test.

It has been shown that forcing transcription through the centromere (CEN) with a galactose-inducible promoter causes detachment of the centromere from the SPB.^48,49^ Cep3-S575 phosphorylation is needed for kinetochore declustering and increased SPB-CEN dynamics in response to DSBs and has, therefore, been suggested to relieve the attachment of centromeres to the SPB,^45^ though the *cep3*^*S575A*^ mutation was not observed to increase CEN distance from either the SPB or NP after DSB induction.^45,50^ We reasoned that if centromere attachment to the SPB was preventing relocation to the NPC in the *cep3*^*S575A*^ mutant, then forcing transcription through the centromere, thereby interrupting that attachment, could allow for relocation of the repeat tract in the *cep3*^*S575A*^ mutant. We integrated the *GAL1* promoter upstream of the centromere on chromosome 6 (*pGAL1-CEN6*),^51^ which is the chromosome containing the (CAG)_130_ tract. Upon galactose induction and transcription through the centromere, the reduced zone 1 occupancy of the CAG locus in the *cep3*^*S575A*^ strain was rescued to normal wild-type levels (Figure 3A). As a control, we tested the effect of inactivation of CEN6 on repeat association with the NPC in a wild-type strain (Figure S1B). In mid-to-late S-phase cells, there was no additional increase in zone 1 occupancy of the chromosome 6 repeat locus to the periphery, supporting that Cep3 phosphorylation and centromere detachment are working in the same pathway. In G1 cells, peripheral association of the tagged chromosome 6 locus increased from ~20% to ~30% upon CEN6 inactivation by transcription (Figure S1B). Therefore, centromere release from the SPB by either transcription through the centromere or constitutive phosphorylation of Cep3 can allow a greater chance of locus-NPC association unrelated to DNA damage, but it is not sufficient for the further increase in repeat-NPC association observed in mid-to-late S phase (Figures 1 and 2), which is dependent on replication through the locus.^2^ We conclude that phosphorylation of Cep3-S575 may allow for relocation of damage resulting from collapsed forks to the NPC by relieving centromere attachment to the SPB.

Detachment from the centromere can increase the mobility of that chromosome.^45^ It is also known that induced DSBs increase both local^52^ and global^53^ chromosome mobility in a mec1-dependent manner (reviewed previously^54^). This DSB-induced mobility was shown to be dependent on Cep3 phosphorylation in one study,^45^ but not another.^50^ We have previously published that the (CAG)_130_ tract, in contrast to a DSB, does not increase either local or global chromosome mobility but instead causes decreased locus mobility relative to chromosomes lacking the repeat tract.^2^ However, those data were collected from unsynchronized cells in S phase, and the locus likely becomes constrained only after anchoring to the NPC. We reasoned that an increase in chromosome mobility due to a collapsed fork at the (CAG)_130_ tract and subsequent Cep3-S575 phosphorylation could be transient. The anchoring at the NPC occurs in a distinct time window in late S phase, between about 50 and 70 min after release from G1 (Figure 1E) and peaking at ~60 min (Figures 1E and 2D)^11^; therefore, any chromosome 6 movement could be confined to a short time period preceding NPC anchoring. The radius of constraint, a measurement of locus mobility,^55^ was measured for the (CAG)_130_ locus in cells that were synchronously released from G1 phase over a 100-min time course segmented into 5-min intervals. During each 5-min interval, images were acquired every 1.5 s, closely mirroring the conditions used previously,^45^ and mean-squared displacement (MSD) curves for each 5-min window were plotted to obtain a radius of constraint. An example of the MSD curve for the first 100 s of the 60-min time point is shown (Figure 3B). For each time point, the radius of constraint was calculated based on the plateau reached within Δt = 100 s (Figure 3C; Table S3). No difference in the mobility of chromosome 6 adjacent to the (CAG)_130_ tract was observed between wild-type and *cep3*^*S575A*^ cells (Figure 3C). Though a fast-directed motion may not have been detected by this method, especially if the timing differed between cells, this result argues against an increase in global chromosome mobility triggered by Cep3 phosphorylation in response to a collapsed fork.

Another driver of DSB mobility is local nucleosome depletion.^50^ Loss of Nhp6, encoded in two loci Nhp6a and Nhp6b, has been shown to reduce histone occupancy on DNA,^56^ and this leads to increased chromosome mobility.^50,57^ However, deletion of Nhp6a and Nhp6b (*nhp6*ΔΔ) did not cause a decrease in relocation, and when combined with *cep3*^*S575A*^, did not rescue the relocation defect of those cells (Figure 3D). Altogether, we conclude that an increase in chromosome mobility does not explain the importance of Cep3 phosphorylation for relocation of the (CAG)_130_ tract to the NP.

Centromeres unattached to the spindle can activate the spindle assembly checkpoint (SAC) via Mad2,^58^ and phosphorylation of Cep3 has been shown to activate the SAC.^45^ Therefore, we tested whether activation of the SAC by Mad2 is required for repositioning; however, there was no relocation defect in *mad2Δ* cells (Figure 3D). Analysis of the DNA content by flow cytometry showed no obvious delays in cell cycle progression in either of the Cep3 mutants (Figure S1C). We conclude that failure of the *cep3*^*S575A*^ mutant to reposition the repeat tract to the NP is not due to a defect in SAC activation or cell cycle progression. Therefore, centromere release appears to be the necessary role of Cep3 phosphorylation.

### Expanded CAG repeat loci associate with DIMs in a Cep3-S575 phosphorylation-dependent manner

**Figure 4.**
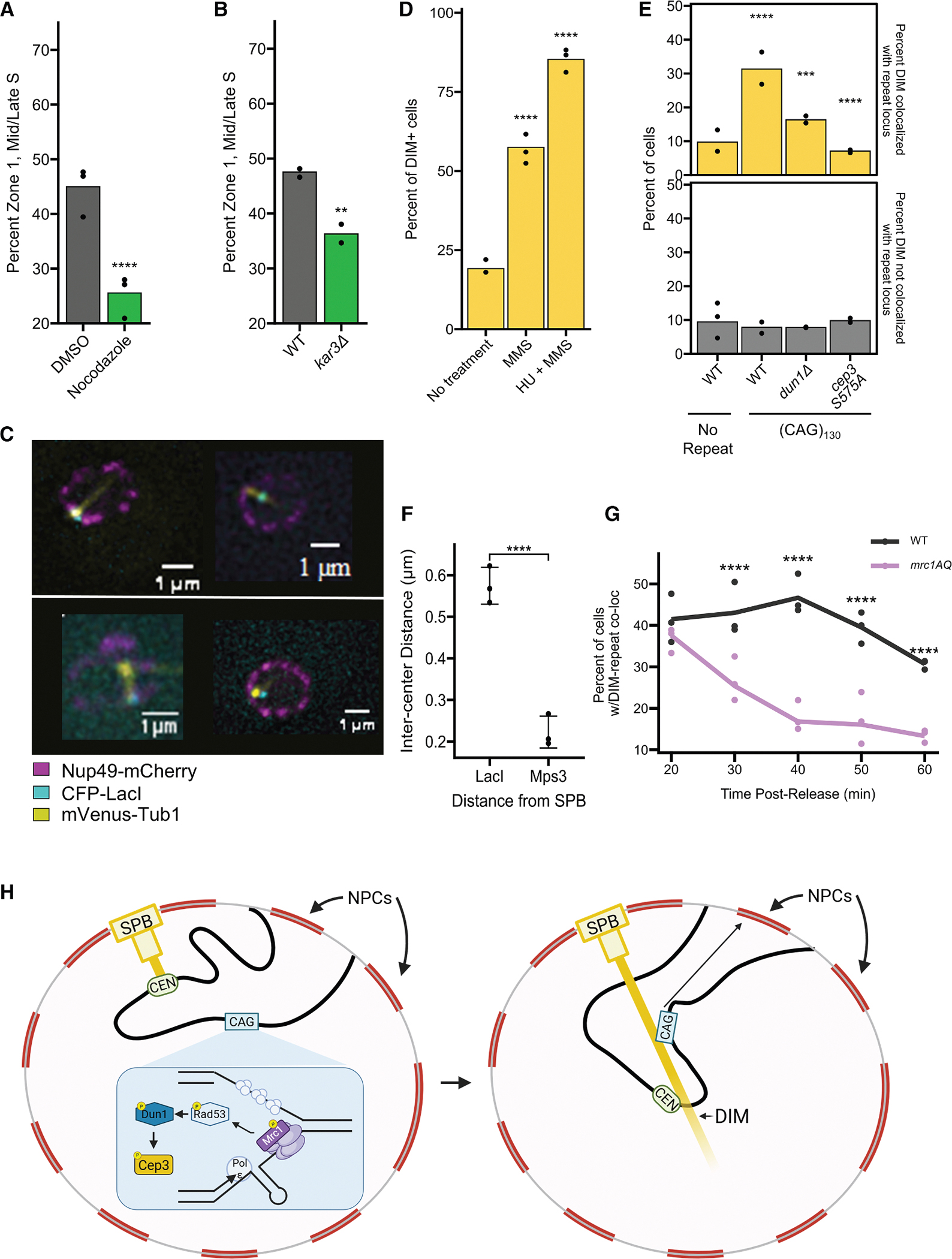
DIMs associate with expanded CAG repeats in a Dun1 and Cep3 phosphorylation-dependent manner (A) Zoning analysis for cells treated with either 15 μg/mL nocodazole or DMSO (0.1%). Points indicate results from 3 biological replicates. (B) Zoning analysis for *kar3Δ*. Zoning performed and analyzed as described in Figure 1. (C) Example images of CFP-LacI colocalizing with DIMs. Scale bars, 1 μm. (D) Percent of mid-to-late S-phase cells (no CAG tract strain) treated as indicated with at least 1 DIM. Points represent results from 3 biological replicates. >275 cells were counted for each condition (avg. 330). Significance calculated compared with no treatment by chi-squared test. (E) Percent of DIM-containing cells of the indicated genotype. DIMs were scored as colocalized or not colocalized with the CFP-LacI focus. Points represent results from at least 2 biologically independent experiments. >140 cells (avg. 202) were counted for each genotype. DIM frequency and colocalization were compared with the wild-type strain by chi-squared test (Table S6). (F) Distance between the SPB (marked with mVenus-Tub1 or mRuby2-Tub1) and CFP-LacI or Mps3-GFP. Mean + SD shown for 3 biological replicates. Comparison by Welch’s t test (Figure S3A; Table S7). (G) Time course data of CFP-LacI (CAG)_130_ colocalized with DIMs. Points are individual results of 3 biological replicates. Statistics were calculated by Bonferroni-corrected chi-squared test (Table S9). *p* values. *p ≤ 0.05, **p ≤ 0.01, ***p ≤ 0.001, ****p ≤ 0.0001 compared with the wild-type (CAG)_130_ strain. (H) Proposed model for the relocation of a structure forming CAG repeat to the NPC. Collapsed or uncoupled forks at a CAG repeat result in Mrc1 phosphorylation, followed by Rad53 activation. Rad53-activated Dun1 leads to the phosphorylation of Cep3. This phosphorylation alters the SPB-CEN attachment, leading to DIM formation. This may allow repositioning of the centromere on the DIM. The repeat locus associates with the DIM to permit relocation of the damaged locus to the NPC. Created with Biorender.com.

In yeast, microtubule structures termed “DIMs” form in response to DSBs in a Rad9 checkpoint-dependent manner.^26^ Interestingly, inactivating a centromere is sufficient to drive the formation of DNA DIMs.^26^ Since our data supported that centromere release by Cep3 phosphorylation was needed to allow relocation of the CAG locus to the NP, we wondered whether DIM formation might be involved. We reasoned that if DIMs were important for relocation to the periphery, then the formation of microtubules in general would be important. To test this, cells were treated for 30 min with either 15 μg/mL of the tubulin depolymerizing drug nocodazole or an equivalent amount of the vehicle (DMSO), and the zoning assay was performed. Addition of nocodazole significantly reduced the percentage of the (CAG)_130_ tract localized to the NP in mid-to-late S-phase cells (Figure 4A). It is known that the minus end-directed microtubule motor Kar3 promotes, though is not strictly required for, DIM formation.^26^ Therefore, we tested whether deletion of Kar3 had an effect on the frequency of (CAG)_130_ locus association with the NP. In *kar3Δ* cells in S phase, relocation of the repeat to zone 1 was reduced (Figure 4B), in line with the reported *kar3Δ* effect on DIMs.

To determine if DIMs were being recruited to the CAG repeat locus, we integrated an additional copy of the tubulin subunit Tub1 tagged with mVenus at the *TUB1* genomic locus.^59^ GFP-LacI was replaced with CFP-LacI and GFP-Nup49 with Nup49-mCherry to allow simultaneous visualization of all 3 nuclear components (Figure 4C). Consistent with previous data,^26^ treatment with 0.03% MMS caused DIM formation in 62% of S-phase cells (Figure 4D). To test whether DIMs were induced by fork collapse, we treated cells with 0.03% MMS + 0.2 M hydroxyurea for 60 min, which caused genome-wide fork collapse and relocation of marked chromosome loci to the NP.^1,11^ Under fork collapsing conditions, 87% of mid-to-late S-phase cells contained a DIM (Figure 4D). We then tested whether a single expanded (CAG)_130_ repeat locus was sufficient to cause DIM formation in cells. We observed that there was an increase in the prevalence of DIM formation in cells with an expanded CAG repeat compared with the no-tract controls (Figure 4E). The majority of these DIMs colocalized with the repeat locus (Figure 4E; see Figures 4C and S2A, for examples). In some cases, we were able to observe the movement of the repeat locus along the DIM from the nuclear interior to the NP (Figure S2B; Video S1). Because DIMs emanate from the SPB, we wanted to know if the repeat locus would be specifically enriched at the SPB. To assess this, we measured the distance between the CFP-LacI foci at the periphery and SPB and compared it to the distance between two SPB markers (Tub1 and Mps3). We found that while the repeat is sometimes found colocalized with the SPB, it is, on average, ~600 nm away, which is significantly different than if the repeat and SPB were colocalized (Figures 4F and S3A). Therefore, we conclude that the repeat locus is not specifically targeted to the SPB.

DIM formation was dependent upon Dun1 and Cep3 phosphorylation, as deletion of Dun1 or mutation to *cep3*^*S575A*^ caused a significant reduction in DIMs (Figure 4E). The repeat tract-induced DIMs were specifically affected in these mutants (Figure 4E). Because *cep3*^*S575A*^ and *kar3Δ* are both involved in this relocation pathway, and had yet to be characterized for CAG repeat fragility, we tested each mutant in a yeast artificial chromosome (YAC) end loss assay.^60^ We found that the *kar3Δ* mutant exhibited increased YAC end loss, but the *cep3*^*S575A*^ mutant did not (Figure S3B). These results are consistent with previously reported DNA repair defects for each protein^45,61^ and suggest that microtubule formation may be more important than centromere release in the ultimate completion of repair.

Finally, to determine the kinetics of DIM formation and whether the formation depended on the DRC, we performed a time course monitoring DIM prevalence in wild-type or *mrc1*^*AQ*^ cells (Figure 4G). This analysis revealed several interesting findings. First, *mrc1*^*AQ*^ mutants have significantly reduced DIM formation, indicating that DIMs are dependent not only on the DDC but also on the DRC. Repeat-associated DIMs are significantly increased in wild-type compared with *mrc1*^*AQ*^ cells starting at 30 min post release from the alpha factor arrest, peak at 40 min, and decline thereafter, though they are still significantly increased out to the 60-min time point. Therefore, checkpoint-dependent DIMs occur both before and coincident with (CAG)_130_ locus relocation to the NPC (Figure 1E). There is also a checkpoint-independent class of microtubule extrusions that occur in early S phase (20 min post release) and decrease thereafter. Altogether, our data show a checkpoint-dependent induction of DIMs and recruitment of a DIM to the site of replication-associated damage to mediate relocation of the damage resulting from collapsed forks to the NPC.

## DISCUSSION

In this study, we aimed to determine if the DDR plays a role in the relocation of structure-forming CAG repeats to the NPC during S phase. We previously showed that long CAG/CTG repeat tracts activate the DDR and that the fork protection complex containing Mrc1 is crucial for preventing CAG fragility and instability and promoting fork progression through the repeat.^16,17,19^ Other systems showed that the Mec1/ATR checkpoint is required for the relocation of chromosomes with difficult-to-repair DNA damage.^1,3,24,25^ In yeast, checkpoint activation by Mec1 (primarily) or Tel1, though not Rad9, is required for repositioning of an HO endonuclease-induced DSB.^1^ Ddc2 is recruited to eroded telomeres with similar timing of repositioning to the nuclear pore,^24^ indirectly suggesting a role for Mec1 in telomere repositioning. In higher eukaryotes, it has been shown that ATR is the primary checkpoint sensor required for repositioning of heterochromatic breaks^25^ and for the recruitment of F-actin in response to aphidicolin.^3^ It was unknown what, if any, role the checkpoint played in the relocation of collapsed forks or other replication-associated damage caused by DNA structures to the NP. Even for types of damage where a requirement for checkpoint proteins had been established, the mechanism by which the checkpoint pathway mediates the repositioning of damaged chromosomal loci had not been elucidated. Although we also found a role for both Mec1 and Tel1/Rad9, we identified phosphorylation of the fork protection component Mrc1 (hClaspin) as the most critical first event that initiates the repositioning program at a DNA structure-induced replication barrier. Furthermore, we elucidated essential phosphorylated targets that allow the damaged locus to move to the NP for repair, defining a role for the replication checkpoint.

Our results indicate that the Mrc1/Rad53 axis is required for the repositioning of CAG repeats to the NPC in mid-to-late S phase. The (CAG)_130_ repeat forms hairpin structures that interfere with DNA replication,^62,63^ and CAG repeats have similar relocation requirements as cells that have been treated with both HU and MMS^1,2,11^; thus, it was speculated that the relocation event was a consequence of a collapsed fork.^2,11^ Since the phosphorylation of Mrc1 occurs in response to uncoupled forks and is critical for the relocation of the expanded repeat to the NP, results presented in this study imply that an uncoupled fork is the initial lesion being sensed and then processed for relocation to the NPC.

We found that there is redundancy in the sensing of the lesion caused by the (CAG)_130_ tract, suggesting that by-products of fork uncoupling such as ssDNA gaps or an exposed DSB end caused by fork reversal or breakage also come into play. A model that fits our data is that the initial event leading to relocation usually begins with Mec1 activating Rad53, with Mrc1 being phosphorylated by a combination of Mec1 and Rad53.^36^ Long-range resection and RPA SUMOylation are required for CAG repeat relocation to the NPC^11^; thus, we would expect RPA-coated ssDNA, which recruits Mec1, to be available prior to relocation. Rad9, which acts at ssDNA-dsDNA junctions to control resection and the accumulation of homologous recombination factors,^64^ and which is activated by both Mec1 and Tel1, was required only when Mec1 and/or the 9–1-1 complex were compromised, indirectly suggesting a secondary role for Tel1. Tel1 has been shown to bind to the MRX complex,^65^ which binds DSB ends and the 3′ end of DNA gaps^66^ and is also required for relocation.^11^ These results imply that either ssDNA or a 3′ end can activate the pathway needed for repositioning, suggesting that there could be a transition from the initial uncoupled fork to a reversed fork or DNA break that provides an end (Figure 1F). It is also possible that a resected gap, which can be bound by 9–1-1,^67^ Rad9,^64^ and Mre11/Tel1, is the secondary signal. This redundancy in signaling leads to a robust mechanism to activate the Rad53 checkpoint in response to the initial fork stall caused by an expanded CAG repeat.

Rad53 is the master regulator of the checkpoint in *S. cerevisiae* and has multiple roles in the response to stalled or collapsed replication forks.^27,68–70^ One well-studied target of Rad53 is the kinase Dun1,^37^ which we determined was needed for repositioning (Figure 2B). It is important to note that while Dun1 is a required target of Rad53 for relocation, it may not be the only target, as the kinase-deficient *rad53*^*K227A*^ mutant is proficient for Dun1 activation^71^ yet is defective in CAG tract repositioning. Indeed, it has been shown that Rad53 phosphorylates Mrc1 in response to fork stalling induced by both hydroxyurea and MMS,^36^ so Mrc1 is likely another critical target of the Rad53 kinase, and there could be others. Dun1 phosphorylates several downstream targets, including the recombination factor Rad55,^37^ RNR inhibition factors (Crt1, Sml1, and Dif1, reviewed in the study by Nordlund and Reichard^38^), and the kinetochore protein Cep3.^45^ Of these targets, the phosphorylation of Cep3 was required for relocation (Figure 2C).

The yeast centromere is only ~120 bp in length and has three sub-sections: CDEI, CDEII, and CDEIII. Kinetochore proteins at each centromere bind to a single microtubule, which is attached to the SPB throughout most of the cell cycle,^72^ with a transient release in early S phase during centromere replication.^73^ Cep3 has a zinc-finger DNA-binding domain that interacts with the CDEIII region as a member of the CBF3 complex (reviewed in the study by Biggins^74^). Phosphorylation of the kinetochore has been widely studied and has varying functions (reviewed in the studies by Funabiki and Wynne and Klemm et al.^75,76^). For example, phosphorylation of kinetochore proteins, such as Dam1, by Aurora B kinase (Ipl1) destabilizes erroneous microtubule attachments in mitosis.^77,78^ However, the consequences of Cep3 phosphorylation have been comparatively poorly described. It was previously suggested that Cep3 phosphorylation led to a release of centromere constraint to allow the increased global mobility of chromosomes observed in response to an HO-induced DSB^45^; however, another group was unable to show a Cep3-dependent increase in the radius of constraint.^50^ Our results indicate that, in response to replication-associated damage, Cep3 phosphorylation may act through another mechanism besides increasing local or global chromatin mobility, namely, to release kinetochore-microtubule attachment and allow DIM formation.

DIMs form in response to DNA damage induced by MMS, zeocin, camptothecin, or I-SceI-induced DSBs.^26^ With this work, we have now shown that while DIM formation is common after treatment with MMS, it is dramatically increased after treatment with HU + MMS, which has been shown to be fork collapsing. DIM formation even occurs as a result of a single fork collapse caused by an expanded CAG repeat tract. Interestingly, much like how CEN inactivation by transcription eliminates the need for Cep3 phosphorylation for relocation, CEN inactivation can cause spontaneous formation of DIMs.^26^ This suggests that while the initial damage (such as an uncoupled fork) is responsible for kinetochore release from the SPB microtubule through Cep3 phosphorylation, the DIM may form as a result of that release rather than directly responding to the damage. Thus, we hypothesize that release of the centromere from the end of the SPB microtubule triggers the formation of a DIM. Supporting this idea, microtubule extrusions can form as a result of centromere replication,^73^ and we observed DIM-like structures that were not dependent on Mrc1 phosphorylation in early S phase (20 min post release in Figure 4G). Therefore, any event that causes release of the centromere from the SPB, be it replication, transcription, or damage-induced Cep3 phosphorylation, may allow a microtubule extrusion, but only the latter category is damage inducible.

The microtubule motor protein Kar3 has been shown to promote the capture and directional movement of damaged loci by DIMs.^26^ When centromeres are inactivated, kinetochores that were previously attached to the microtubule plus end reposition and bind the microtubule side-on.^79,80^ Kar3, as a minus-end-directed motor, is involved in repositioning the centromere on the microtubule by hindering tethering of the kinetochore to the plus end of the microtubule.^79–81^ During this kinetochore release and repositioning process, the damaged repeat locus could be freed up to move to the NPC. If this is the case, then we might expect that the presence of Kar3 could increase the duration of this “relocation permissive” state by antagonizing the reattachment of the kinetochore to the microtubule plus end, which could explain why a *kar3Δ* reduces but does not completely abrogate relocation to the NPC. What remains unclear is whether the microtubule interacts directly with the repeat-induced lesion or if the DIM allows for repositioning of the locus in an indirect manner, for example, by allowing free movement of the damaged locus after kinetochore release or allowing the centromere to slide along the DIM. The increased co-localization of the repeat locus with the DIM suggests the possibility of a direct role (Figure 4E). It has recently been shown that DIMs serve as sites of repair center nucleation, with liquid droplets of Rad52 colocalizing with DIMs.^82^ Interestingly, the kinetics of DIM association with the CAG repeat (Figure 4G) strongly resemble the previously described kinetics of Rad52 recruitment to the repeat locus.^2^ Rad52^2^ and RPA^11^ are recruited to the site of the (CAG)_130_ tract prior to relocation; these factors become SUMOylated and are required for relocation to the NPC through an interaction with Slx5/8^11^. Slx5/8, in turn, is critical for the anchoring of damaged DNA at the NPC (reviewed previously^8^). SUMOylation has also been shown to promote the condensation of repair factors.^83^ Possibly, SUMOylation or factors within SUMOylation-induced repair centers could play a role in the association between the collapsed fork at the CAG tract and the DIM.

Thus, we propose that after fork uncoupling at the CAG repeat, the DRC results in the phosphorylation of Cep3. This phosphorylation causes centromere release from the SPB, which promotes DIM formation. The DIM allows movement of the collapsed fork (or damage resulting from the uncoupled fork) to the periphery in a manner yet to be fully elucidated but that may involve interaction of the damaged locus with the DIM. Once at the periphery, interactions between SUMOylated repair factors, Slx5/8, and the nucleoporins tether the locus to the NPC for repair or fork restart (Figure 4H).

Previous data have shown that loss of Mec1, Rad9, 9–1-1 loading, Mrc1, or Rad53 checkpoint competency lead to increased fragility of the CAG repeat,^17,19^ and we found that loss of Kar3 also significantly increased the loss of a chromosome end distal to a (CAG)_130_ repeat. However, there does not appear to be a tight correlation between the level of CAG repeat fragility and relocation defects, as the *mrc1*^*AQ*^ and *cep3*^*S575A*^ mutants, which are relocation deficient, had little effect on chromosome end loss (this work and previous work from our lab^19^). Based on previous data using other repair assays that do not require chromosome end loss,^6,84^ defects in NPC relocation can change repair choice or repair fidelity but may not necessarily lead to failed repair.

There is prior evidence in other types of eukaryotic cells for a physical mechanism by which the movement of lesions to repair centers within the nucleus occurs. Heterochromatic DSBs in *Drosophila* require nuclear F-actin and myosin for directed movement to the NP.^85^ In human cells, the relocation of forks stressed by aphidicolin treatment is dependent on the polymerization of nuclear F-actin, and PCNA foci were observed to move along actin filaments to the NP.^3^ It has been shown that the recruitment of nuclear actin in response to damage can control pathway choice in response to replication stress.^86^ It is unclear if yeast have equivalent nuclear actin filaments or if directed movement to the NP would be detectable in the smaller yeast nucleus. However, in yeast, the kinesin-14 motor protein complex is required for the movement of telomeric DSBs from their usual attachment site on the nuclear membrane to NPCs, and these were observed to move along DIMs.^26,61^ Our results suggest that collapsed forks may also utilize DIMs to facilitate their movement to the yeast NPC. In human cells, it was recently reported that cytoplasmic microtubules that form in response to DSBs push on the nuclear envelope to form nuclear envelope tubules (NETs) that elongate into the nucleus and bring the nuclear envelope into the proximity of DSBs within the nuclear interior, and the DSBs may also move toward the dsbNETs.^87^ The formation of these dsbNETs serves to mobilize repair proteins to the sites of DSBs and is dependent on the ATM/ATR/DNA-PK checkpoint response.^87^ Thus, the involvement of microtubules in mobilizing a repair response seems to be conserved, though the details of where in the nucleus the DIMs form and how they interact with the damaged locus may differ between organisms. It will be interesting to elucidate if DIMs are utilized in multicellular eukaryotic cells or if their function has been replaced by a combination of nuclear actin filaments and dsbNETs in these larger nuclei.

In summary, our results illuminate an additional role for the DRC and DDC response, which is to phosphorylate a kinetochore component in response to fork uncoupling to allow for repositioning of the resulting damaged locus within the nucleus. Since this repositioning is important for repair, we identify it as a crucial role for the DDC response in *S. cerevisiae*. Repositioning of damaged loci to a repair center is present across many organisms. Where it has been studied, the checkpoint is critical to facilitate this relocation, but the targets involved have not been elucidated. Given the importance of recovery from replication fork collapse, a mechanism to allow centromere release to facilitate damaged locus repositioning might be especially critical at regions with fork-stalling DNA structures and for cancerous or aging cells experiencing high levels of replication stress.

### Limitations of the study

Rad53 has a large number of targets that have been previously identified, and this study only examined a subset of potential targets for their effect on CAG repeat-induced repositioning. Though we have identified Mrc1 as an initial sensor in this pathway, implicating an uncoupled fork as the initial form of damage, we do not know the state of the DNA upon anchoring to the periphery, which occurs up to 20 min after the initial signal that initiates DIM formation. Finally, because most experiments were microscopy based, we do not yet have a clear picture of how the DIMs interact with the repeat locus (or associated proteins) or how the DIMs facilitate repositioning of the locus to the NPC.

### RESOURCE AVAILABILITY

#### Lead contact

Requests for information and resources should be directed to the lead contact, Catherine H. Freudenreich (catherine.freudenreich@tufts.edu).

#### Materials availability

Reagents generated in this study will be made available upon reasonable request.

#### Data and code availability

- Data associated with this manuscript can be found in the provided supplemental tables.
- This paper does not report original code.
- Any additional information required to reanalyze the data reported in this paper is available from the lead contact upon request.

## STAR★METHODS

### EXPERIMENTAL MODEL AND STUDY PARTICIPANT DETAILS

#### Yeast strains and genetic manipulation

Standard procedures were used in cell growth and medium preparation. All *S. cerevisiae* strains were made in the W303 (RAD5+) or BY4705 background and are listed in Table S10. Point mutant alleles *rad53*^*K227A*^,^94^ *cep3*^*S575A*45^ have been previously characterized and were generated using CRISPR-Cas9 methods as described previously.^88^ See Table S9 for primers used to create each mutation. Correct mutation was screened by both restriction enzyme digest and sequencing. The *P*_*GAL1*_*-CEN6* construct has been previously characterized.^51^ Centromere inactivation was confirmed by plating cells onto galactose containing media and observing a decrease in viability compared to glucose. Venus-Tub1 was introduced as an additional copy using plasmids pHIS3p:Venus-Tub1+3′UTR:: LEU2, or pHIS3p:mRuby2-Tub1+3′UTR::HPH, which were gifts from Wei-Lih Lee.^59^

### METHOD DETAILS

#### Microscopy

Colonies were checked for 130 CAG repeat units by PCR with primers flanking the repeat (CTGRev2/T720 or 2StepCAG_pAG32-F/R). PCR products separated on a 2% agarose gel (MetaPhor, Lonza). Cells from colonies with the correct repeat length were inoculated into YC media, grown overnight at 30°C with shaking, diluted back to OD = 0.2 and grown to approximately 5 × 10^6^ cells per mL in YC media. Cells were fixed with 4% paraformaldehyde and washed 3 times with 1× PBS. 1.4% YC agar was used to make agar pads on depression slides and 5 μL of cells were added on top with a coverslip. z stack images were taken using a Zeiss AX10 fluorescent microscope and/or a Delta Vision Ultra High-Resolution using an Olympus 100× with 1.45NA. Step interval size was 0.15–0.2 μm and approximately 25 Z-planes were taken per field of cells. Exposure time was approximately DIC: 100ms; GFP: 300ms and mCherry: 500ms (for strains with mCherry-TetR). Images were deconvolved, and three-zoning criteria was used to evaluate the location of the GFP foci for mid/late S-phase cells with the ImageJ point picker program as described in.^95^ Only the middle two-thirds of the stacks were used for analysis. Mid/Late S-phase cells were determined by yeast morphology using bud size criteria of 2/3^rd^ or less, and at least 15% the size of the mother cell.^96^ For drug treatments, cells were grown to approximately 1 × 10^7^, split into 2 cultures and grown for 30 min in either 0.1% dimethyl sulfoxide (DMSO) or 15 μg/mL nocodazole. The cells were then fixed and imaged as described above. For DIM experiments, cells were prepared as described above and imaged with the filters (and exposure): Cyan (200ms), Yellow (200ms), Red (200ms). Cells were scored for colocalization determined by any overlap (not inclusive of “touching”) of the repeat and the nuclear periphery, and of the repeat with DIMs. DIMs were defined as monopolar microtubule structures emanating from the SPB during mid-late S phase (See Figure 4C for examples).

#### *P*_*GAL1*_-CEN6 inactivation

Colonies were checked for 130 CAG repeats by PCR with primers flanking the repeat (CTGRev2/T720). Cells from colonies with the correct repeat length were grown overnight in synthetic complete media +2% glucose. Cultures were spun down and resuspended in YEP-lactate media to an OD_600_ of 0.3 and grown overnight. Cultures were spun down, split in half, and resuspended in synthetic complete media +2% galactose or +2% glucose to an OD_600_ of 0.3. Cultures were grown for 3 h followed by the zoning assay protocol as outlined above.

#### MSD analysis

Colonies were checked for 130 CAG repeats by PCR with primers flanking the repeat (2StepCAG_pAG32-F/R). Cells with correct repeat length were grown to approximately OD_600_ of 0.5 before being transferred to a concanavalin A-coated chamber slide for attachment. From there, cells were synchronized by addition of alpha factor (1 μM final concentration) then released by washing 3× with YPD media. A single field of cells was imaged with DeltaVision Ultra high resolution microscope using a 100× objective using optical axis integration every 1.5s for 5 min. This was repeated using cells in different areas of the slide until a total of 100 min of timepoints were taken. Exposure was: Red 400ms, Green 400ms, Pol 50ms (every 50^th^ frame). Images were analyzed with CellProfiler, tracking the LacO/LacI-mCherry focus and correcting for nuclear translation by tracking Nup49-GFP. Tracked foci were quantified using MSD=<〖(x⇀−(x_dt)⇀)〗^2> and radiusofconstraint=sqrt(5/4×MSDplateau) (Dion & Gasser, 2013). The plateau was calculated by regression in Graphpad PRISM software using the first 100 Δt timepoints.

#### Fragility analysis

Method adapted from previous work^11^ with modifications. Cells containing a yeast artificial chromosome with a (CAG)_130_ repeat were grown to colonies on YC-Ura-Leu and selected for correct tract length. For an experiment, single cells were spread on YC-Leu and grown into colonies to a standard size (typically 3 days) to allow breakage of the YAC within the colony during growth. These colonies were then checked for tract length to rule out ones in which the repeat had detectably contracted or expanded. 10 colonies were selected from each of these plates, resuspended in water, and a dilution was plated on 1g/L FOA media lacking leucine. Additionally, a dilution of cells was plated on YC-Leu to quantify total cells plated. Cells were grown to colonies, counted, and rates of breakage were calculated using the MSS method of maximum likelihood using RSalvador.^91^ *p value*s were calculated using the log likelihood method in RSalvador.^97^

#### Flow cytometry analysis

Flow cytometry was done similar as previously.^98^ Briefly, cells were grown overnight in YPD media, diluted to an OD_600_ of 0.2 and grown to log phase. Cells were arrested by addition of 1 μM Alpha Factor (Zymo). Cells were released into YPD ~1 × 10^7^ cells were collected and fixed and permeabilized in 70% ethanol overnight. Fixed cells were incubated with 0.4mg/mL RNaseA overnight at 37C. Cells were then treated with 1mg/mL Proteinase K at 50C for 30 min. Cells were stained with 1μM Sytox Green and run on an Attune NxT. The data were analyzed using openCyto.^99^

### QUANTIFICATION AND STATISTICAL ANALYSIS

Prism software was used to calculate statistical significance. For microscopy experiments the chi-squared test was used. Graphs were generated using Prism, or R (using ggprism^100^) as appropriate. For all zoning experiments, an average of ~100 cells were analyzed for each strain isolate (range 48–170) totaling an average of ~200 cells (range 122–378) for every genotype. For colocalization experiments, an average of ~100 cells were counted per experiment per timepoint per strain (range 39–148), with 2–3 experimental replicates performed, for a total of ~200–300 cells per condition. For DIM experiments an average of ~100 cells were counted per experiment (range 63–159), with 2–3 experimental replicates performed, for a total of ~200–300 cells per condition. Replicates were biologically independent experiments consisting of two independent isolates for mutants. For all other experiment types, at least 3 biologically independent experiments were performed, done with at least two independent isolates for mutants. *p-value*s. (*) *p* ≤ 0.05, (**) *p* ≤ 0.01 (***) *p* ≤ 0.001, (****) *p* ≤ 0.0001 compared with wild-type (CAG)_130_ strain by chi-squared test. (^^^^) *p* ≤ 0.0001 compared as indicated by chi-squared test. For YAC end loss, at least 3 experiments consisting of at least 9 colonies per genotype, error bars are 95% confidence interval, *p value*s are calculated by the log likelihood method in RSalvador. (^^^^) *p* ≤ 0.0001 compared as indicated.

## Supplementary Material

1

2

3

Supplemental information can be found online at https://doi.org/10.1016/j.celrep.2025.116083.

## Figures and Tables

**KEY RESOURCES TABLE:** 

REAGENT or RESOURCE	SOURCE	IDENTIFIER

Chemicals, peptides, and recombinant proteins		

Concanavalin A	Themo Fisher Scientific	AAJ61221MD
5-Fluoroorotic Acid Monohydrate	USBiological	F5050
MetaPhor^®^ Agarose	Lonza	50180
Paraformaldehyde	Fisher Scientific	AC41678-0250
Dimethyl Sulfoxide	Fisher Scientific	BP231-100
Hydroxyurea	Fisher Scientific	AC151681000
Methyl Methane Sulfonate	Fisher Scientific	AC15689-0050
Alpha Factor Mating Hormone	Zymo Research	Y1001
RNaseA	Thermo Fisher Scientific	EN0531
Proteinase K	Thermo Fisher Scientific	EO0491
SyTox Green	Thermo Fisher Scientific	S7020

Experimental models: Organisms/strains		

See Table S11 for a list of yeast strains used in this study. Backgrounds include BY4705 or W303	Freudenreich Lab	Strain number

Oligonucleotides		

See Table S10 for a list of oligonucleotides used in this study.	Eton Bioscience Inc.	Primer name

Recombinant DNA		

pHIS3p:Venus-Tub1+3′UTR::LEU2	Markus et al.^59^	CHF837 Addgene 50644
pHIS3p:mRuby2-Tub1+3′UTR::HPH	Markus et al.^59^	CHF956 Addgene 50633
pRCC-N	Generoso et al.^88^	CHF709 Addgene 81192
pAO139 mrc1AQ LEU2	Osborn et al.^30^	CHF614
pFA6a-KANMX6	Longtine et al.^89^	CHF136
pYM-N35	Janke et al.^90^	CHF397

Software and Algorithms		

RSalvador	Zheng^91^	https://github.com/eeeeeric/rSalvador
ImageJ (FIJI)	Schindelin et al.^92^	https://imagej.net/software/fiji/
CellProfiler	Stirling et al.^93^	https://github.com/CellProfiler
Graphpad PRISM Sotware version 10		www.graphpad.com

## Highlights

- The DNA replication checkpoint triggers repositioning of CAG/CTG tracts to the NPC
- Mrc1 phosphorylation requirement implicates fork uncoupling as the checkpoint signal
- Cep3 phosphorylation allows centromere release and is critical for NPC association
- Damage-inducible microtubules (DIMs) are required for repeat relocation to the NPC

## References

[1] Nagai S, Dubrana K, Tsai-Pflugfelder M, Davidson MB, Roberts TM, Brown GW, Varela E, Hediger F, Gasser SM, and Krogan NJ (2008). Functional Targeting of DNA Damage to a Nuclear Pore–Associated SUMO-Dependent Ubiquitin Ligase. Science 322, 597–602. 10.1126/science.1162790. 18948542 PMC3518492

[2] Su XA, Dion V, Gasser SM, and Freudenreich CH (2015). Regulation of recombination at yeast nuclear pores controls repair and triplet repeat stability. Genes Dev. 29, 1006–1017. 10.1101/gad.256404.114. 25940904 PMC4441049

[3] Lamm N, Read MN, Nobis M, Van Ly D, Page SG, Masamsetti VP, Timpson P, Biro M, and Cesare AJ (2020). Nuclear F-actin counteracts nuclear deformation and promotes fork repair during replication stress. Nat. Cell Biol. 22, 1460–1470. 10.1038/s41556-020-00605-6. 33257806

[4] Horigome C, Unozawa E, Ooki T, and Kobayashi T (2019). Ribosomal RNA gene repeats associate with the nuclear pore complex for maintenance after DNA damage. PLoS Genet. 15, e1008103. 10.1371/journal.pgen.1008103. 30998688 PMC6490929

[5] Kramarz K, Schirmeisen K, Boucherit V, Ait Saada A, Lovo C, Palancade B, Freudenreich C, and Lambert SAE (2020). The nuclear pore primes recombination-dependent DNA synthesis at arrested forks by promoting SUMO removal. Nat. Commun. 11, 5643. 10.1038/s41467-020-19516-z. 33159083 PMC7648084

[6] Aguilera P, Whalen J, Minguet C, Churikov D, Freudenreich C, Simon M-N, and Géli V (2020). The nuclear pore complex prevents sister chromatid recombination during replicative senescence. Nat. Commun. 11, 160. 10.1038/s41467-019-13979-5. 31919430 PMC6952416

[7] Pinzaru AM, Kareh M, Lamm N, Lazzerini-Denchi E, Cesare AJ, and Sfeir A (2020). Replication stress conferred by POT1 dysfunction promotes telomere relocalization to the nuclear pore. Genes Dev. 34, 1619–1636. 10.1101/gad.337287.120. 33122293 PMC7706707

[8] Whalen JM, and Freudenreich CH (2020). Location, Location, Location: The Role of Nuclear Positioning in the Repair of Collapsed Forks and Protection of Genome Stability. Genes 11, 635. 10.3390/genes11060635. 32526925 PMC7348918

[9] Lamm N, Rogers S, and Cesare AJ (2021). Chromatin mobility and relocation in DNA repair. Trends Cell Biol. 31, 843–855. 10.1016/j.tcb.2021.06.002. 34183232

[10] Gasser SM, and Stutz F (2023). SUMO in the regulation of DNA repair and transcription at nuclear pores. FEBS Lett. 597, 2833–2850. 10.1002/1873-3468.14751. 37805446

[11] Whalen JM, Dhingra N, Wei L, Zhao X, and Freudenreich CH (2020). Relocation of Collapsed Forks to the Nuclear Pore Complex Depends on Sumoylation of DNA Repair Proteins and Permits Rad51 Association. Cell Rep. 31, 107635. 10.1016/j.celrep.2020.107635. 32402281 PMC7344339

[12] Usdin K, House NCM, and Freudenreich CH (2015). Repeat instability during DNA repair: Insights from model systems. Crit. Rev. Biochem. Mol. Biol. 50, 142–167. 10.3109/10409238.2014.999192. 25608779 PMC4454471

[13] Khristich AN, and Mirkin SM (2020). On the wrong DNA track: Molecular mechanisms of repeat-mediated genome instability. J. Biol. Chem. 295, 4134–4170. 10.1074/jbc.REV119.007678. 32060097 PMC7105313

[14] Scully R, Walter JC, and Nussenzweig A (2024). One-ended and two-ended breaks at nickase-broken replication forks. DNA Repair 144, 103783. 10.1016/j.dnarep.2024.103783. 39504607 PMC11681922

[15] Triplett MK, Johnson MJ, and Symington LS (2024). Induction of homologous recombination by site-specific replication stress. DNA Repair 142, 103753. 10.1016/j.dnarep.2024.103753. 39190984 PMC11425181

[16] Sundararajan R, and Freudenreich CH (2011). Expanded CAG/CTG Repeat DNA Induces a Checkpoint Response That Impacts Cell Proliferation in Saccharomyces cerevisiae. PLoS Genet. 7, e1001339. 10.1371/journal.pgen.1001339. 21437275 PMC3060079

[17] Lahiri M, Gustafson TL, Majors ER, and Freudenreich CH (2004). Expanded CAG Repeats Activate the DNA Damage Checkpoint Pathway. Mol. Cell 15, 287–293. 10.1016/j.molcel.2004.06.034. 15260979

[18] Freudenreich CH, and Lahiri M (2004). Structure-Forming CAG/CTG Repeat Sequences are Sensitive to Breakage in the Absence of Mrc1 Checkpoint Function and S-Phase Checkpoint Signaling: Implications for Trinucleotide Repeat Expansion Diseases. Cell Cycle 3, 1370–1374. 10.4161/cc.3.11.1246. 15483399

[19] Gellon L, Kaushal S, Cebrián J, Lahiri M, Mirkin SM, and Freudenreich CH (2019). Mrc1 and Tof1 prevent fragility and instability at long CAG repeats by their fork stabilizing function. Nucleic Acids Res. 47, 794–805. 10.1093/nar/gky1195. 30476303 PMC6344861

[20] Hustedt N, Gasser SM, and Shimada K (2013). Replication Checkpoint: Tuning and Coordination of Replication Forks in S Phase. Genes 4, 388–434. 10.3390/genes4030388. 24705211 PMC3924824

[21] McClure AW, Canal B, and Diffley JFX (2022). A DNA replication fork-centric view of the budding yeast DNA damage response. DNA Repair 119, 103393. 10.1016/j.dnarep.2022.103393. 36108423

[22] Katou Y, Kanoh Y, Bando M, Noguchi H, Tanaka H, Ashikari T, Sugimoto K, and Shirahige K (2003). S-phase checkpoint proteins Tof1 and Mrc1 form a stable replication-pausing complex. Nature 424, 1078–1083. 10.1038/nature01900. 12944972

[23] Lou H, Komata M, Katou Y, Guan Z, Reis CC, Budd M, Shirahige K, and Campbell JL (2008). Mrc1 and DNA Polymerase ϵ Function Together in Linking DNA Replication and the S Phase Checkpoint. Mol. Cell 32, 106–117. 10.1016/j.molcel.2008.08.020. 18851837 PMC2699584

[24] Khadaroo B, Teixeira MT, Luciano P, Eckert-Boulet N, Germann SM, Simon MN, Gallina I, Abdallah P, Gilson E, Géli V, and Lisby M (2009). The DNA damage response at eroded telomeres and tethering to the nuclear pore complex. Nat. Cell Biol. 11, 980–987. 10.1038/ncb1910. 19597487

[25] Chiolo I, Minoda A, Colmenares SU, Polyzos A, Costes SV, and Karpen GH (2011). Double-Strand Breaks in Heterochromatin Move Outside of a Dynamic HP1a Domain to Complete Recombinational Repair. Cell 144, 732–744. 10.1016/j.cell.2011.02.012. 21353298 PMC3417143

[26] Oshidari R, Strecker J, Chung DKC, Abraham KJ, Chan JNY, Damaren CJ, and Mekhail K (2018). Nuclear microtubule filaments mediate non-linear directional motion of chromatin and promote DNA repair. Nat. Commun. 9, 2567. 10.1038/s41467-018-05009-7. 29967403 PMC6028458

[27] Zhao X, Muller EG, and Rothstein R (1998). A Suppressor of Two Essential Checkpoint Genes Identifies a Novel Protein that Negatively Affects dNTP Pools. Mol. Cell 2, 329–340. 10.1016/S1097-2765(00)80277-4. 9774971

[28] Fay DS, Sun Z, and Stern DF (1997). Mutations in SPK1/RAD53 that specifically abolish checkpoint but not growth-related functions. Curr. Genet. 31, 97–105. 10.1007/s002940050181. 9021124

[29] Naylor ML, Li J.m., Osborn AJ, and Elledge SJ (2009). Mrc1 phosphorylation in response to DNA replication stress is required for Mec1 accumulation at the stalled fork. Proc. Natl. Acad. Sci. USA 106, 12765–12770. 10.1073/pnas.0904623106. 19515819 PMC2722297

[30] Osborn AJ, and Elledge SJ (2003). Mrc1 is a replication fork component whose phosphorylation in response to DNA replication stress activates Rad53. Genes Dev. 17, 1755–1767. 10.1101/gad.1098303. 12865299 PMC196183

[31] Emili A (1998). MEC1-Dependent Phosphorylation of Rad9p in Response to DNA Damage. Mol. Cell 2, 183–189. 10.1016/S1097-2765(00)80128-8. 9734355

[32] Gilbert CS, Green CM, and Lowndes NF (2001). Budding Yeast Rad9 Is an ATP-Dependent Rad53 Activating Machine. Mol. Cell 8, 129–136. 10.1016/S1097-2765(01)00267-2. 11511366

[33] House NCM, Yang JH, Walsh SC, Moy JM, and Freudenreich CH (2014). NuA4 Initiates Dynamic Histone H4 Acetylation to Promote High-Fidelity Sister Chromatid Recombination at Postreplication Gaps. Mol. Cell 55, 818–828. 10.1016/j.molcel.2014.07.007. 25132173 PMC4169719

[34] House NC, Polleys EJ, Quasem I, De la Rosa Mejia M, Joyce CE, Takacsi-Nagy O, Krebs JE, Fuchs SM, and Freudenreich CH (2019). Distinct roles for S. cerevisiae H2A copies in recombination and repeat stability, with a role for H2A.1 threonine 126. eLife 8, e53362. 10.7554/eLife.53362. 31804179 PMC6927750

[35] Bacal J, Moriel-Carretero M, Pardo B, Barthe A, Sharma S, Chabes A, Lengronne A, and Pasero P (2018). Mrc1 and Rad9 cooperate to regulate initiation and elongation of DNA replication in response to DNA damage. EMBO J. 37, e99319. 10.15252/embj.201899319. 30158111 PMC6213276

[36] Alcasabas AA, Osborn AJ, Bachant J, Hu F, Werler PJ, Bousset K, Furuya K, Diffley JF, Carr AM, and Elledge SJ (2001). Mrc1 transduces signals of DNA replication stress to activate Rad53. Nat. Cell Biol. 3, 958–965. 10.1038/ncb1101-958. 11715016

[37] Bashkirov VI, Bashkirova EV, Haghnazari E, and Heyer W-D (2003). Direct Kinase-to-Kinase Signaling Mediated by the FHA Phosphoprotein Recognition Domain of the Dun1 DNA Damage Checkpoint Kinase. Mol. Cell Biol. 23, 1441–1452. 10.1128/MCB.23.4.1441-1452.2003. 12556502 PMC141154

[38] Nordlund P, and Reichard P (2006). Ribonucleotide Reductases. Annu. Rev. Biochem. 75, 681–706. 10.1146/annurev.biochem.75.103004.142443. 16756507

[39] de la Torre Ruiz M-A, and Lowndes NF (2000). DUN1 defines one branch downstream of RAD53 for transcription and DNA damage repair in Saccharomyces cerevisiae. FEBS Lett. 485, 205–206. 10.1016/S0014-5793(00)02198-0. 11186433

[40] Zhou Z, and Elledge SJ (1993). DUN1 encodes a protein kinase that controls the DNA damage response in yeast. Cell 75, 1119–1127. 10.1016/0092-8674(93)90321-G. 8261511

[41] Fortin GS, and Symington LS (2002). Mutations in yeast Rad51 that partially bypass the requirement for Rad55 and Rad57 in DNA repair by increasing the stability of Rad51–DNA complexes. EMBO J. 21, 3160–3170. 10.1093/emboj/cdf293. 12065428 PMC126052

[42] Huang M, Zhou Z, and Elledge SJ (1998). The DNA Replication and Damage Checkpoint Pathways Induce Transcription by Inhibition of the Crt1 Repressor. Cell 94, 595–605. 10.1016/S0092-8674(00)81601-3. 9741624

[43] Wu X, and Huang M (2008). Dif1 Controls Subcellular Localization of Ribonucleotide Reductase by Mediating Nuclear Import of the R2 Subunit. Mol. Cell Biol. 28, 7156–7167. 10.1128/MCB.01388-08. 18838542 PMC2593381

[44] Zhao X, and Rothstein R (2002). The Dun1 checkpoint kinase phosphorylates and regulates the ribonucleotide reductase inhibitor Sml1. Proc. Natl. Acad. Sci. 99, 3746–3751. 10.1073/pnas.062502299. 11904430 PMC122595

[45] Strecker J, Gupta GD, Zhang W, Bashkurov M, Landry M-C, Pelletier L, and Durocher D (2016). DNA damage signalling targets the kinetochore to promote chromatin mobility. Nat. Cell Biol. 18, 281–290. 10.1038/ncb3308. 26829389

[46] Lechner J, and Carbon J (1991). A 240 kd multisubunit protein complex, CBF3, is a major component of the budding yeast centromere. Cell 64, 717–725. 10.1016/0092-8674(91)90501-o. 1997204

[47] Lechner J (1994). A zinc finger protein, essential for chromosome segregation, constitutes a putative DNA binding subunit of the Saccharomyces cerevisiae kinetochore complex, Cbf3. EMBO J. 13, 5203–5211. 10.1002/j.1460-2075.1994.tb06851.x. 7957085 PMC395469

[48] Collins KA, Castillo AR, Tatsutani SY, and Biggins S (2005). De Novo Kinetochore Assembly Requires the Centromeric Histone H3 Variant. Mol. Biol. Cell 16, 5649–5660. 10.1091/mbc.E05-08-0771. 16207811 PMC1289410

[49] Hill A, and Bloom K (1987). Genetic manipulation of centromere function. Mol. Cell Biol. 7, 2397–2405. 3302676 10.1128/mcb.7.7.2397-2405.1987 PMC365371

[50] Cheblal A, Challa K, Seeber A, Shimada K, Yoshida H, Ferreira HC, Amitai A, and Gasser SM (2020). DNA Damage-Induced Nucleosome Depletion Enhances Homology Search Independently of Local Break Movement. Mol. Cell 80, 311–326.e4. 10.1016/j.molcel.2020.09.002. 32970994

[51] Pobiega S, and Marcand S (2010). Dicentric breakage at telomere fusions. Genes Dev. 24, 720–733. 10.1101/gad.571510. 20360388 PMC2849128

[52] Dion V, Kalck V, Horigome C, Towbin BD, and Gasser SM (2012). Increased mobility of double-strand breaks requires Mec1, Rad9 and the homologous recombination machinery. Nat. Cell Biol. 14, 502–509. 10.1038/ncb2465. 22484486

[53] Miné-Hattab J, and Rothstein R (2012). Increased chromosome mobility facilitates homology search during recombination. Nat. Cell Biol. 14, 510–517. 10.1038/ncb2472. 22484485

[54] García Fernández F, and Fabre E (2022). The Dynamic Behavior of Chromatin in Response to DNA Double-Strand Breaks. Genes 13, 215. 10.3390/genes13020215. 35205260 PMC8872016

[55] Dion V, and Gasser SM (2013). Chromatin Movement in the Maintenance of Genome Stability. Cell 152, 1355–1364. 10.1016/j.cell.2013.02.010. 23498942

[56] Celona B, Weiner A, Di Felice F, Mancuso FM, Cesarini E, Rossi RL, Gregory L, Baban D, Rossetti G, Grianti P, (2011). Substantial Histone Reduction Modulates Genomewide Nucleosomal Occupancy and Global Transcriptional Output. PLoS Biol. 9, e1001086. 10.1371/journal.pbio.1001086. 21738444 PMC3125158

[57] Hauer MH, Seeber A, Singh V, Thierry R, Sack R, Amitai A, Kryzhanovska M, Eglinger J, Holcman D, Owen-Hughes T, and Gasser SM (2017). Histone degradation in response to DNA damage enhances chromatin dynamics and recombination rates. Nat. Struct. Mol. Biol. 24, 99–107. 10.1038/nsmb.3347. 28067915

[58] Hoyt MA, Totis L, and Roberts BT (1991). S. cerevisiae genes required for cell cycle arrest in response to loss of microtubule function. Cell 66, 507–517. 10.1016/0092-8674(81)90014-3. 1651171

[59] Markus SM, Omer S, Baranowski K, and Lee W-L (2015). Improved Plasmids for Fluorescent Protein Tagging of Microtubules in Saccharomyces cerevisiae. Traffic Cph. Den. 16, 773–786. 10.1111/tra.12276. PMC4795465 25711127

[60] Polleys EJ, and Freudenreich CH (2018). Methods to Study Repeat Fragility and Instability in Saccharomyces cerevisiae. In Genome Instability: Methods and Protocols, Muzi-Falconi M and Brown GW, eds. (Springer), pp. 403–419. 10.1007/978-1-4939-7306-4_28. 29043639

[61] Chung DKC, Chan JNY, Strecker J, Zhang W, Ebrahimi-Ardebili S, Lu T, Abraham KJ, Durocher D, and Mekhail K (2015). Perinuclear tethers license telomeric DSBs for a broad kinesin- and NPC-dependent DNA repair process. Nat. Commun. 6, 7742. 10.1038/ncomms8742. 26205667

[62] Pelletier R, Krasilnikova MM, Samadashwily GM, Lahue R, and Mirkin SM (2003). Replication and Expansion of Trinucleotide Repeats in Yeast. Mol. Cell Biol. 23, 1349–1357. 10.1128/MCB.23.4.1349-1357.2003. 12556494 PMC141142

[63] Viterbo D, Michoud G, Mosbach V, Dujon B, and Richard G-F (2016). Replication stalling and heteroduplex formation within CAG/CTG trinucleotide repeats by mismatch repair. DNA Repair 42, 94–106. 10.1016/j.dnarep.2016.03.002. 27045900

[64] Ferrari M, Rawal CC, Lodovichi S, Vietri MY, and Pellicioli A (2020). Rad9/53BP1 promotes DNA repair via crossover recombination by limiting the Sgs1 and Mph1 helicases. Nat. Commun. 11, 3181. 10.1038/s41467-020-16997-w. 32576832 PMC7311424

[65] Cassani C, Gobbini E, Wang W, Niu H, Clerici M, Sung P, and Longhese MP (2016). Tel1 and Rif2 Regulate MRX Functions in End-Tethering and Repair of DNA Double-Strand Breaks. PLoS Biol. 14, e1002387. 10.1371/journal.pbio.1002387. 26901759 PMC4762649

[66] Seppa IM, Ceppi I, Tennakoon M, Reginato G, Jackson J, Rouault CD, Agashe S, Sviderskiy VO, Limbu M, Lantelme E, (2025). MRN–CtIP, EXO1, and DNA2–WRN/BLM act bidirectionally to process DNA gaps in PARPi-treated cells without strand cleavage. Genes Dev. 39, 582–602. 10.1101/gad.352421.124. 40127955 PMC12047661

[67] Zheng F, Georgescu RE, Yao NY, O’Donnell ME, and Li H (2023). Structures of 9–1-1 DNA checkpoint clamp loading at gaps from start to finish and ramification on biology. Cell Rep. 42, 112694. 10.1016/j.celrep.2023.112694. 37392384 PMC10529453

[68] Santocanale C, and Diffley JF (1998). A Mec1- and Rad53-dependent checkpoint controls late-firing origins of DNA replication. Nature 395, 615–618. 10.1038/27001. 9783589

[69] Herzberg K, Bashkirov VI, Rolfsmeier M, Haghnazari E, McDonald WH, Anderson S, Bashkirova EV, Yates JR, and Heyer W-D (2006). Phosphorylation of Rad55 on Serines 2, 8, and 14 Is Required for Efficient Homologous Recombination in the Recovery of Stalled Replication Forks. Mol. Cell Biol. 26, 8396–8409. 10.1128/MCB.01317-06. 16966380 PMC1636779

[70] McClure AW, and Diffley JF (2021). Rad53 checkpoint kinase regulation of DNA replication fork rate via Mrc1 phosphorylation. eLife 10, e69726. 10.7554/eLife.69726. 34387546 PMC8387023

[71] Hoch NC, Chen ES-W, Buckland R, Wang S-C, Fazio A, Hammet A, Pellicioli A, Chabes A, Tsai M-D, and Heierhorst J (2013). Molecular basis of the essential s phase function of the rad53 checkpoint kinase. Mol. Cell Biol. 33, 3202–3213. 10.1128/MCB.00474-13. 23754745 PMC3753913

[72] Heun P, Laroche T, Raghuraman MK, and Gasser SM (2001). The Positioning and Dynamics of Origins of Replication in the Budding Yeast Nucleus. J. Cell Biol. 152, 385–400. 11266454 10.1083/jcb.152.2.385 PMC2199623

[73] Kitamura E, Tanaka K, Kitamura Y, and Tanaka TU (2007). Kinetochore–microtubule interaction during S phase in Saccharomyces cerevisiae. Genes Dev. 21, 3319–3330. 10.1101/gad.449407. 18079178 PMC2113032

[74] Biggins S (2013). The Composition, Functions, and Regulation of the Budding Yeast Kinetochore. Genetics 194, 817–846. 10.1534/genetics.112.145276. 23908374 PMC3730914

[75] Funabiki H, and Wynne DJ (2013). Making an effective switch at the kinetochore by phosphorylation and dephosphorylation. Chromosoma 122, 135–158. 10.1007/s00412-013-0401-5. 23512483 PMC3665160

[76] Klemm C, Thorpe PH, and Ólafsson G (2021). Cell-cycle phosphoregulation of the kinetochore. Curr. Genet. 67, 177–193. 10.1007/s00294-020-01127-2. 33221975

[77] Tanaka TU, Rachidi N, Janke C, Pereira G, Galova M, Schiebel E, Stark MJR, and Nasmyth K (2002). Evidence that the Ipl1-Sli15 (Aurora kinase-INCENP) complex promotes chromosome bi-orientation by altering kinetochore-spindle pole connections. Cell 108, 317–329. 10.1016/s0092-8674(02)00633-5. 11853667

[78] Pinsky BA, Kung C, Shokat KM, and Biggins S (2006). The Ipl1-Aurora protein kinase activates the spindle checkpoint by creating unattached kinetochores. Nat. Cell Biol. 8, 78–83. 10.1038/ncb1341. 16327780

[79] Tanaka K, Kitamura E, Kitamura Y, and Tanaka TU (2007). Molecular mechanisms of microtubule-dependent kinetochore transport toward spindle poles. J. Cell Biol. 178, 269–281. 10.1083/jcb.200702141. 17620411 PMC2064446

[80] Tanaka TU, Stark MJR, and Tanaka K (2005). Kinetochore capture and bi-orientation on the mitotic spindle. Nat. Rev. Mol. Cell Biol. 6, 929–942. 10.1038/nrm1764. 16341079

[81] Tytell JD, and Sorger PK (2006). Analysis of kinesin motor function at budding yeast kinetochores. J. Cell Biol. 172, 861–874. 10.1083/jcb.200509101. 16533946 PMC2063730

[82] Oshidari R, Huang R, Medghalchi M, Tse EYW, Ashgriz N, Lee HO, Wyatt H, and Mekhail K (2020). DNA repair by Rad52 liquid droplets. Nat. Commun. 11, 695. 10.1038/s41467-020-14546-z. 32019927 PMC7000754

[83] Keiten-Schmitz J, Schunck K, and Müller S (2020). SUMO Chains Rule on Chromatin Occupancy. Front. Cell Dev. Biol. 7, 343. 10.3389/fcell.2019.00343. 31998715 PMC6965010

[84] Horigome C, Bustard DE, Marcomini I, Delgoshaie N, Tsai-Pflugfelder M, Cobb JA, and Gasser SM (2016). PolySUMOylation by Siz2 and Mms21 triggers relocation of DNA breaks to nuclear pores through the Slx5/Slx8 STUbL. Genes Dev. 30, 931–945. 10.1101/gad.277665.116. 27056668 PMC4840299

[85] Caridi CP, D’Agostino C, Ryu T, Zapotoczny G, Delabaere L, Li X, Khodaverdian VY, Amaral N, Lin E, Rau AR, and Chiolo I (2018). Nuclear F-actin and myosins drive relocalization of heterochromatic breaks. Nature 559, 54–60. 10.1038/s41586-018-0242-8. 29925946 PMC6051730

[86] Palumbieri MD, Merigliano C, González-Acosta D, Kuster D, Krietsch J, Stoy H, von Känel T, Ulferts S, Welter B, Frey J, (2023). Nuclear actin polymerization rapidly mediates replication fork remodeling upon stress by limiting PrimPol activity. Nat. Commun. 14, 1–15. 10.1038/s41467-023-43183-5. 38016948 PMC10684888

[87] Shokrollahi M, Stanic M, Hundal A, Chan JNY, Urman D, Jordan CA, Hakem A, Espin R, Hao J, Krishnan R, (2024). DNA double-strand break–capturing nuclear envelope tubules drive DNA repair. Nat. Struct. Mol. Biol. 31, 1319–1330. 10.1038/s41594-024-01286-7. 38632359

[88] Generoso WC, Gottardi M, Oreb M, and Boles E (2016). Simplified CRISPR-Cas genome editing for Saccharomyces cerevisiae. J. Microbiol. Methods 127, 203–205. 10.1016/j.mimet.2016.06.020. 27327211

[89] Longtine MS, McKenzie A, Demarini DJ, Shah NG, Wach A, Brachat A, Philippsen P, and Pringle JR (1998). Additional modules for versatile and economical PCR-based gene deletion and modification in Saccharomyces cerevisiae. Yeast 14, 953–961. 10.1002/(SICI)1097-0061(199807)14:10<953::AID-YEA293>3.0.CO;2-U. 9717241

[90] Janke C, Magiera MM, Rathfelder N, Taxis C, Reber S, Maekawa H, Moreno-Borchart A, Doenges G, Schwob E, Schiebel E, and Knop M (2004). A versatile toolbox for PCR-based tagging of yeast genes: new fluorescent proteins, more markers and promoter substitution cassettes. Yeast 21, 947–962. 10.1002/yea.1142. 15334558

[91] Zheng Q (2017). rSalvador: An R Package for the Fluctuation Experiment. G3 (Bethesda). 7, 3849–3856. 10.1534/g3.117.300120. 29084818 PMC5714482

[92] Schindelin J, Arganda-Carreras I, Frise E, Kaynig V, Longair M, Pietzsch T, Preibisch S, Rueden C, Saalfeld S, Schmid B, (2012). Fiji: an open-source platform for biological-image analysis. Nat. Methods 9, 676–682. 10.1038/nmeth.2019. 22743772 PMC3855844

[93] Stirling DR, Swain-Bowden MJ, Lucas AM, Carpenter AE, Cimini BA, and Goodman A (2021). CellProfiler 4: improvements in speed, utility and usability. BMC Bioinf. 22, 433. 10.1186/s12859-021-04344-9. PMC8431850 34507520

[94] Pike BL, Hammet A, and Heierhorst J (2001). Role of the N-terminal Forkhead-associated Domain in the Cell Cycle Checkpoint Function of the Rad53 Kinase*210. J. Biol. Chem. 276, 14019–14026. 10.1074/jbc.M009558200. 11278522

[95] Meister P, Gehlen LR, Varela E, Kalck V, and Gasser SM (2010). Chapter 21 - Visualizing Yeast Chromosomes and Nuclear Architecture. In Methods in Enzymology Guide to Yeast Genetics: Functional Genomics, Proteomics, and Other Systems Analysis (Academic Press), pp. 535–567. 10.1016/S0076-6879(10)70021-5. 20946824

[96] Hediger F, Neumann FR, Van Houwe G, Dubrana K, and Gasser SM (2002). Live Imaging of Telomeres: yKu and Sir Proteins Define Redundant Telomere-Anchoring Pathways in Yeast. Curr. Biol. 12, 2076–2089. 10.1016/S0960-9822(02)01338-6. 12498682

[97] Zheng Q (2016). Comparing mutation rates under the Luria–Delbrück protocol. Genetica 144, 351–359. 10.1007/s10709-016-9904-3. 27188462

[98] Masnovo C, Paleiov Z, Dovrat D, Baxter LK, Movafaghi S, Aharoni A, and Mirkin SM (2024). Stabilization of expandable DNA repeats by the replication factor Mcm10 promotes cell viability. Nat. Commun. 15, 10532. 10.1038/s41467-024-54977-6. 39627228 PMC11615337

[99] Finak G, Frelinger J, Jiang W, Newell EW, Ramey J, Davis MM, Kalams SA, De Rosa SC, and Gottardo R (2014). OpenCyto: An Open Source Infrastructure for Scalable, Robust, Reproducible, and Automated, End-to-End Flow Cytometry Data Analysis. PLoS Comput. Biol. 10, e1003806. 10.1371/journal.pcbi.1003806. 25167361 PMC4148203

[100] A ggplot2 Extension Inspired by GraphPad Prism https://csdaw.github.io/ggprism/.

