# Supplementary material for: The DNA replication checkpoint targets the kinetochore to reposition DNA structure-induced replication damage to the nuclear periphery
Source: Cell Rep. Author manuscript; Available in PMC 2025 Sep 15. (PMC12435773; doi:10.1016/j.celrep.2025.116083)
Supplement: 1 [file NIHMS2107273-supplement-1.pdf]

Cell Reports, Volume *44*

Supplemental information

The DNA replication checkpoint targets the kinetochore to reposition DNA structure-induced replication damage to the nuclear periphery

Tyler M. Maclay, Jenna M. Whalen, Matthew J. Johnson, and Catherine H. Freudenreich

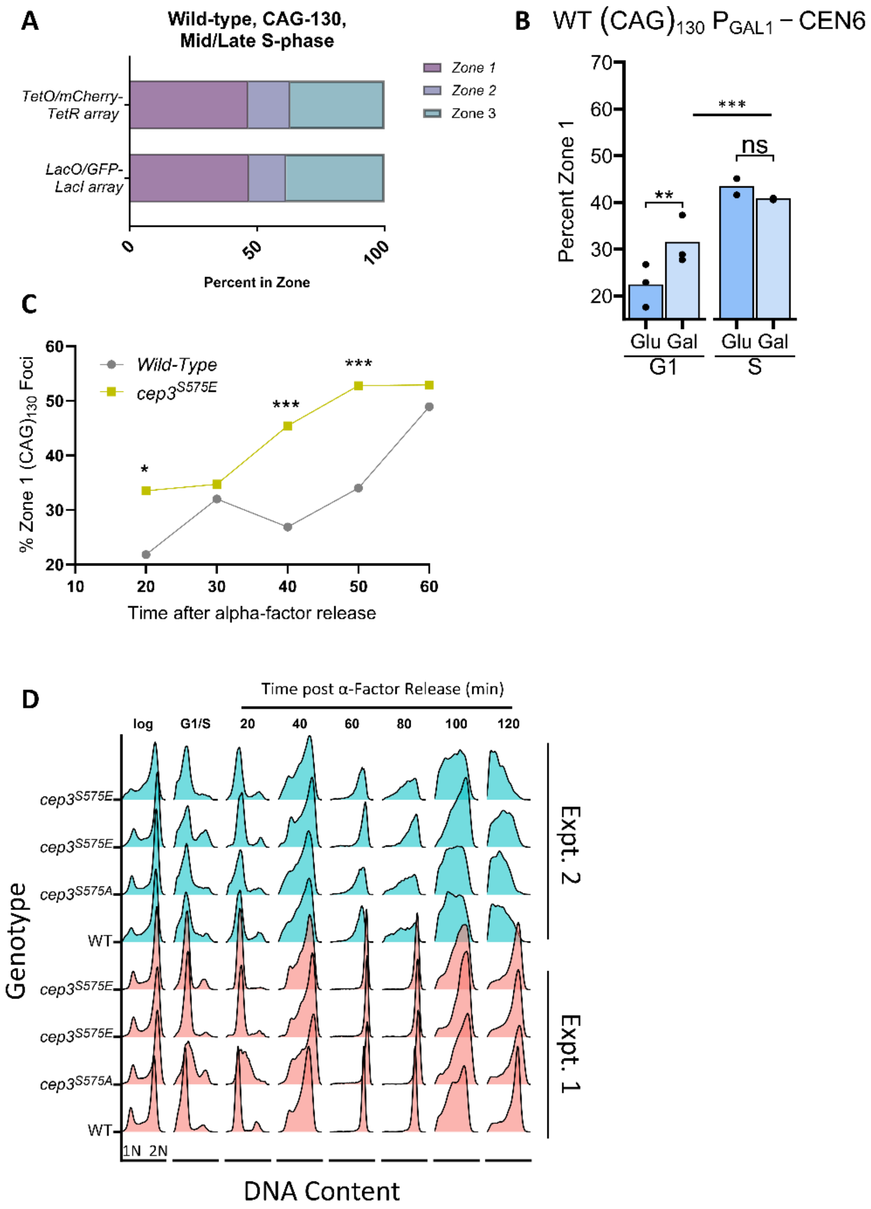

Figure S1: Zoning and cell cycle analyses of WT and strains with functional modulation of the centromere. Percent of zone 1 foci for wild-type mid/late S-phase cells containing the LacO/GFP-LacI array (strain 2744) or TetO/mCherry-TetR array (strain 3116) near ARS0607, ~6.4kb away from a $(CAG)_{130}$ tract inserted into chromosome 6 (see Table S1). B) Zoning assay results for WT cells containing a *GAL1* promoter adjacent to *CEN6* (P_{GAL1}-CEN6) at the indicated cell cycle phase and grown with the indicated sugar (see Table S1 for number of cells analyzed per experiment, zone 1 percentages and p values) . C) Zoning analysis of WT and *cep3-S575E* cells after alpha factor release; >130 cells analyzed per strain per time point (see Table S4 for details). D) DNA content flow cytometry analysis of cells with the indicated genotype, for either log phase cells (1N and 2N DNA content marked), alpha factor-arrested cells arrested at the G1/S boundary, or cells collected after

release into S phase at the indicated timepoints. Two separately run experiments shown. (*) P≤0.05, (**) P≤0.01 (***) P≤0.001, (****) P≤0.0001 compared as indicated by chi-squared test

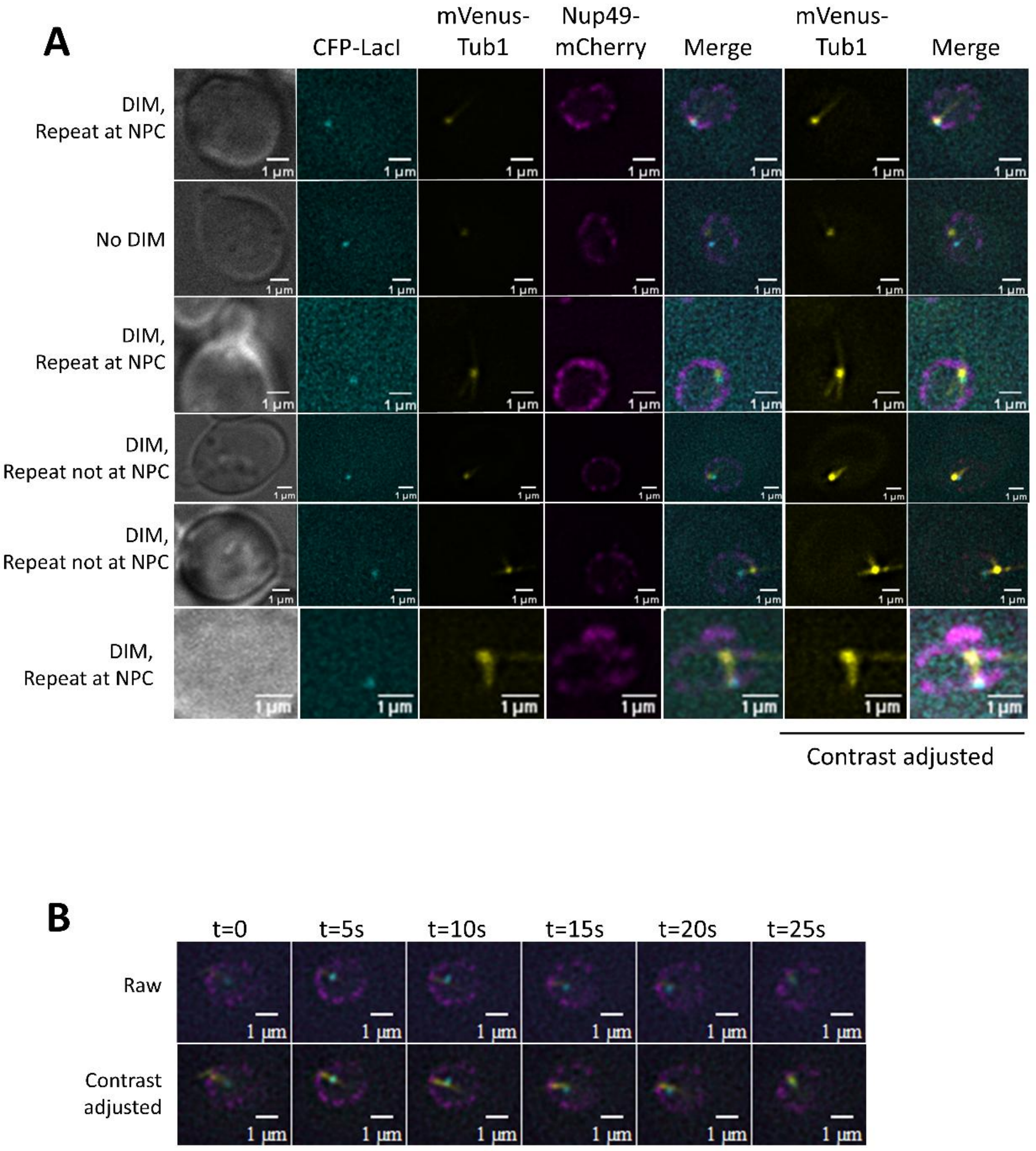

Figure S2: DIM examples: A) Example images of damage-inducible microtubules (DIMs). Defined as monopolar microtubule extrusions during S-phase which exhibit rapidly decaying fluorescence intensity. Cell lacking a DIM shown for context. B) Example frames from a movie of a CFP-LacI focus associating with a DIM and moving toward the NP. Video acquired at 0.2 frame/s. (see also Movie S1) Scale bar is 1µm for all images.

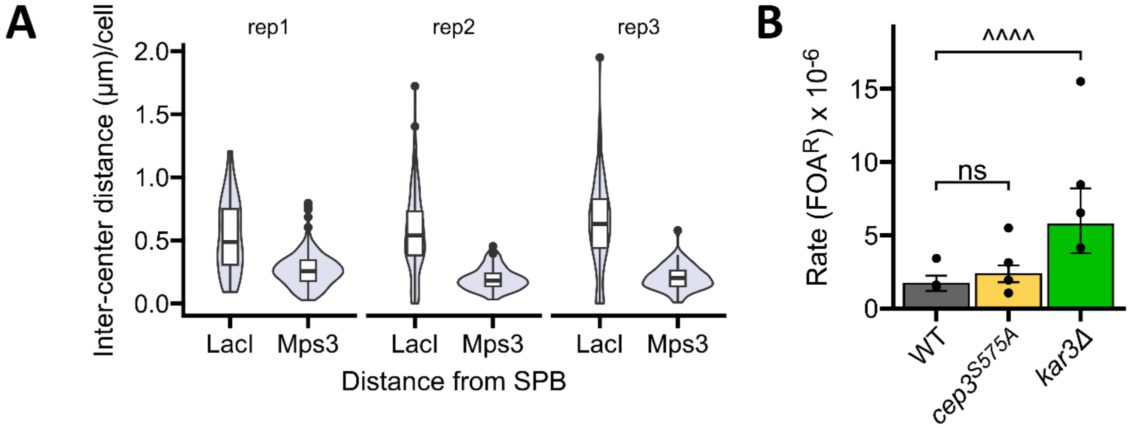

Figure S3: DIM characteristics and consequences for chromosomal fragility:
A) Distribution of inter-centroid distances between the marked pairs (i.e. CFP-LacI/mVenus-Tub1 or Mps3-GFP/mRuby2-Tub1). B) Results from $(CAG)_{130}$ YAC end loss assay. Points represent the calculated rate of FOA^R of experiments consisting of at least 9 colonies, error bars are 95% confidence interval, p values are calculated by the log likelihood method in RSalvador (Table S8).

Table S1: Zoning assay data for the GFP-LacI or mCherry-TetR/$(CAG)_{130}$ locus

Genotype	Strain#	Zone 1[1]	# Cells	Total # Cells	Percent Zone 1	Percent Zone 1 (Total)	P value (Chi Sq. Compared to WT)
WT	2744[2]	78	162	265	48.15	47.55	N/A
	5800[2]	48	103		46.5		
	3116[3]	73	157	157	46.60		
slx5Δ[4]	2797	46	152	152	30.26	30.26	N/A
chk1Δ	5133	43	107	209	40.19	37.32	0.0324
	5134	35	102		34.31		
rad53Δsml1Δ	5203	29	95	150	30.53	30	0.000711
	5219	16	55		29.09		
rad53K227A	4952	24	88	163	27.27	30.06	0.000517
	4953	25	75		33.33		
mec1Δsml1Δ	3447	73	152	210	48.03	61.43	0.00349
	3447	56	58		96.55		
ddc1Δ	4214	56	128	231	43.75	46.32	0.855
	4215	51	103		49.51		
rad24Δ	4120	22	48	122	45.83	43.44	0.52
	4121	31	74		41.89		
rad9Δ	3762	58	122	228	47.54	46.05	0.81
	3763	47	106		44.34		
tel1Δ	3582	34	75	150	45.33	45.33	0.74
	3583	34	75		45.33		
mec1Δrad9Δsml1Δ	4913	27	74	154	36.49	35.06	0.017
	4926	27	80		33.75		
rad9Δrad24Δ	4096	39	120	221	32.50	33.94	0.00327
	4097	36	101		35.64		
mec1Δrad9Δ rad24Δsml1Δ	5093	17	80	160	21.25	24.38	0.00000338
	5094	22	80		27.50		
rad9Δtel1Δ	5193	34	75	152	45.33	47.37	1
	5194	38	77		49.35		
rad9Δrad24Δtel1Δ	5211	22	76	151	28.95	32.45	0.00378
	5212	27	75		36.00		
dun1Δ	5135	37	111	212	33.33	31.13	0.000402
	5136	29	101		28.71		
rad55Δ	5198	26	60	165	43.33	46.67	0.937
	5199	22	50		44.00		
	5200	29	55		52.73		
rad55Δrad57Δ	5226	40	74	152	54.05	53.95	0.248
	5227	42	78		53.85		

sml1Δ	5201	26	51	154	50.98	47.40	1
	5202	47	103		45.63		
dun1Δsml1Δ	5305	32	74	177	43.24	37.29	0.242
	5306	34	103		33.01		
cep3S575A	5277	25	76	174	32.89	33.33	0.00432
	5287	33	98		33.67		
cep3S575E	5393	60	103	164	58.25	57.93	0.09
	5395	35	61		57.38		
dun1Δcep3S575E	5379	53	103	154	51.46	55.19	0.00000652 (Compared to dun1Δ)
	5380	32	51		62.75		
cep3-S575A pGAL1-CEN6 -Galactose	5402	39	125	193	31.20	30.05	N/A
	5403	19	68		27.94		
cep3-S575A pGAL1-CEN6 +Galactose	5402	51	120	179	42.50	48.04	0.000554 (Compared to -Galactose)
	5403	35	59		59.32		
nhp6aΔ	5297	34	75	193	45.33	45.08	0.668
	5298	53	118		44.92		
nhp6bΔ	5318	43	79	154	54.43	50.65	0.609
	5319	35	75		46.67		
nhp6aΔnhp6bΔ	5300	43	93	156	46.24	43.59	0.493
	5301	25	63		39.68		
mrc1Δ+pMRC1-AQ	5618	46	161	331	28.57	31.12	0.00006
	5619	57	170		33.53		
mad2Δ	5616	50	111	251	45.05	49.00	0.808
	5617	73	140		52.14		
kar3Δ	5757	35	92	193	38.04	36.27	0.0207
	5758	35	101		34.65		
WT + DMSO	3116, rep 1	76	162	378	46.91	44.97	N/A
	3116, rep 2	51	107		47.66		
	3116, rep 3	43	109		39.45		
WT +15ug/mL Nocodazole	3116, rep 1	39	144	372	27.08	25.54	0.0000000399 (Compared to DMSO)
	3116, rep 2	33	118		27.97		
	3116, rep 3	23	110		20.91		
WT (CAG-130 ChrIV)	6320	45	102	203	44.12	42.86	N/A
	6321	42	101		41.58		

cep3-S575A (CAG-130 ChrIV)	6353	30	100	201	30.00	30.85	0.0165 (Compared to WT, ChrIV)
	6354	32	101		31.68		
nhp6aΔnhp6bΔ cep3-S575A	5748	30	105	205	28.57	25.37	0.112 (Compared to cep3-S575A)
	5749	22	100		22.00		
WT pGAL1-CEN6 +Glucose G1	5396	27	101	314	26.73	22.29	N/A
	5397	19	108		17.59		
	5398	24	105		22.86		
WT pGAL1-CEN6 +Galactose G1	5396	41	110	211	37.27	31.43	.015 (Comparted to +Glucose)
	5397	28	101		27.72		
	5398	30	104		28.85		
WT pGAL1-CEN6 +Glucose mid/late S	5396	46	102	203	45.10	43.35	N/A
	5397	42	101		41.58		
WT pGAL1-CEN6 +Galactose mid/late S	5396	43	105	211	40.95	40.76	.666 (Compared to +Glucose) <.0001 (Compared to G1 +Galactose)
	5397	43	106		40.57		

[1]The nuclear periphery used to determine the zone was marked with GFP-Nup49

[2]Wild type LacO strain

[3]Wild type TetO strain

[4]Reproduced from Su et al. 2015

Table S2: Colocalization assay time course analysis of CFP-LacI/$(CAG)_{130}$ locus and mCherry-Nup49

Genotype	Time point	No. Co-loc	# Cells	Total # Cells	Percent Co-loc	Percent Co-loc (Total)	adj. P value[2]
WT	20	37	105	323	35.24	38.08	1
		49	118		41.53		
		37	100		37.00		
mrc1AQ		27	92	221	29.35	31.22	
		13	39		33.33		
		29	90		32.22		
WT	30	38	109	328	34.86	35.67	0.68
		40	118		33.90		
		39	101		38.61		
mrc1AQ		25	100	264	25.00	29.55	
		11	40		27.50		
		42	124		33.87		
WT	40	36	119	346	30.25	34.97	1
		53	126		42.06		
		32	101		31.68		
mrc1AQ		36	90	244	40.00	36.89	
		16	41		39.02		
		38	113		33.63		
WT	50	48	118	334	40.68	47.01	0.004
		60	116		51.72		
		49	100		49.00		
mrc1AQ		32	96	255	33.33	32.16	
		14	46		30.43		
		36	113		31.86		
WT	60	55	109	339	50.46	50.44	<.0001
		66	128		51.56		
		50	102		49.02		
mrc1AQ		25	94	248	26.60	30.24	
		15	48		31.25		
		35	106		33.02		
WT	70	54	115	369	46.96	46.07	0.018
		60	127		47.24		
		56	127		44.09		
mrc1AQ		35	93	244	37.63	33.61	
		13	51		25.49		
		34	100		34.00		
WT	80	40	120	381	33.33	35.17	1
		50	138		36.23		

		44	123		35.77		
mrc1AQ		32	98	249	32.65	34.54	
		16	50		32.00		
		38	101		37.62		
WT	90	39	118	378	33.05	33.33	1
		47	142		33.10		
		40	118		33.90		
mrc1AQ		40	112	260	35.71	36.15	
		18	49		36.73		
		36	99		36.36		

[2]Bonferroni-adjusted Chi Sq.

Table S3: Colocalization assay time course analysis, mCherry-TetR/$(CAG)_{130}$ locus and GFP-Nup49

Timepoint	Genotype	No. Co-loc	# Cells	Total # Cells	Percent Co-loc	Percent Co-loc (Total)	adj. P value[2]
10	WT	34	121	245	28.10	30.61	1
		41	124		33.06		
	cep3S575E	38	120	250	31.67	30.40	
		38	130		29.23		
20	WT	37	131	251	28.24	27.09	0.58
		31	120		25.83		
	cep3S575E	39	116	243	33.62	34.16	
		44	127		34.65		
30	WT	37	145	263	25.52	27.00	1
		34	118		28.81		
	cep3S575E	38	113	237	33.63	32.91	
		40	124		32.26		
40	WT	34	121	230	28.10	29.13	0.01
		33	109		30.28		
	cep3S575E	52	123	254	42.28	43.31	
		58	131		44.27		
50	WT	62	148	251	41.89	43.03	1
		46	103		44.66		
	cep3S575E	44	114	229	38.60	40.61	
		49	115		42.61		
60	WT	52	124	226	41.94	46.02	1
		52	102		50.98		
	cep3S575E	45	108	220	41.67	42.27	
		48	112		42.86		

[2]Bonferroni-adjusted Chi sq.

Table S4: Zoning assay time course analysis of mCherry-TetR/$(CAG)_{130}$ locus and GFP-Nup49

			Strain	
			**Wild-type**	**cep3^{S575E}**
Time after alpha-factor release	20	No. Zone 1 foci	44	51
		%	21.8	33.6
		Total No. cells	202	152
		p-value to WT*	----	0.02
	30	No. Zone 1 foci	57	52
		%	32	34.7
		Total No. cells	179	150
		p-value to WT*	----	0.63
	40	No. Zone 1 foci	47	59
		%	26.9	45.4
		Total No. cells	175	130
		p-value to WT*	----	0.001
	50	No. Zone 1 foci	71	93
		%	34	52.8
		Total No. cells	206	176
		p-value to WT*	----	0.0004
	60	No. Zone 1 foci	89	80
		%	48.9	52.9
		Total No. cells	182	151
		p-value to WT*	----	0.51

*using the Fisher's exact test

Table S5: Mean Square Displacement (MSD) analysis of TetR $(CAG)_{130}$ locus

	cep3-S575A			Wild-Type		
min post G1 release	Radius of constraint (μm)*	SEM	N	Radius of constraint (μm)*	SEM	N
5	0.6599	0.07012	5	0.746492	0.06649	9
10	0.9039	0.1217	4	0.619274	0.1205	6
15	0.8986	0.1078	3	0.966566	0.06725	3
20	0.7989	0.2769	2	0.700625	0.07287	5
25	0.9523	0.1002	5	0.631862	0.07965	7
30	0.8444	0.1185	6	0.689656	0.09995	3
35	0.5941	0.08926	6	0.652495	0.08602	8
40	0.8201	0.07469	8	0.707284	0.04335	7
45	0.8821	0.06559	5	0.729812	0.07197	14
50	0.7688	0.1222	8	0.745822	0.04515	8
55	0.8986	0.07013	12	0.744228	0.06408	7
60	0.778	0.0777	8	0.735102	0.07607	5
65	0.6851	0.06772	3	0.732462	0.03682	5
70	1.045	0.1012	4	0.649808	0.1075	4
75	0.7842	0.08749	3	0.618971	0.06715	10
80	0.9161	0.1144	3	0.700179	0.06497	7
85	0.5469	0	1	0.677311	0.2221	2
90	0.6527	0.1263	3	0.809398	0.05053	3
95	0.6576	0.06694	6	0.6257	0.06277	4
100	0.8234	0.06687	4	0.693001	0.07622	4

*calculated from the first Δt =[0,100] seconds of the 5 minute interval shown; cells were imaged every 1.5 seconds.

Table S6: Damage-Inducible Microtubule (DIM) data. Colocalization between CFP-LacI(CAG)$_{130}$, mVenus-Tub1, and mCherry-Nup49

Geno-type	Strain #	DIM at Repeat	DIM not at Repeat	No DIM	# Cells	Total # Cells	% DIM+	% DIM+ Total	% DIM coloc w/ repeat locus	% DIM not coloc w/ repeat locus	% DIM coloc w/ repeat locus (Total)	% DIM not coloc w/ repeat locus (Total)	P value (Chi Sq. compared to WT, CAG-130)
WT, No repeat	5805	20	7	123	150	350	18.00	19.14	13.33	4.67	9.71	9.43	<.0001
		7	15	78	100		22.00		7.00	15.00			
		7	11	82	100		18.00		7.00	11.00			
0.03% MMS	5805	52	46	61	159	362	61.64	57.46					<.0001 (Compared to WT, No repeat)
		19	35	49	103		52.43						
		24	32	44	100		56.00						
0.03% MMS + 0.2M HU	5805	38	52	12	102	278	88.24	85.25					<.0001 (Compared to WT, No repeat)
		37	45	19	101		81.19						
		39	26	10	75		86.67						
WT, CAG-130	5779	40	14	95	149	281			26.85	9.40	31.32	7.83	N/A
		48	8	76	132				36.36	6.06			
dun1Δ	5806	11	5	47	63	141			17.46	7.94	16.31	7.80	0.003
	5807	12	6	60	78				15.38	7.69			
cep3-S575A	5920	8	10	90	108	184			7.41	9.26	7.07	9.78	<.0001
	5921	5	8	63	76				6.58	10.53			

Table S7: SPB-Foci distances

Distance Measured	Replicate	Average	Standard Deviation	n	P value (T test Welch's Correction)
Mps3-SPB	1	0.267	0.134	252	p=.0005
	2	0.196	0.090	108	
	3	0.206	0.100	61	
LacI-SPB (LacI near (CAG)130 locus)	1	0.534	0.294	52	
	2	0.567	0.342	72	
	3	0.622	0.358	103	

Table S8: YAC end loss rates

Strain	Genotype	Rate (x10^-6)	Rate (combined, x10^-6)	95% CI (x10^-6)	P value (log likelihood, compared to WT)
5486	WT	1.49		1.2-2.24	NA
		1.61			
		3.42	1.68		
6337	*cep3-s575a*	1.06		1.79-2.95	0.114
		5.49			
6338		1.94			
		3.13	2.34		
6340	*kar3Δ*	15.50		3.78-8.2	<.0001
		6.53			
		5.79			
6341		4.14			
		8.46	5.73		

Table S9: Damage-Inducible Microtubule Time Course Analysis. Colocalization between CFP-LacI(CAG)$_{130}$, mVenus-Tub1, and mCherry-Nup49

Geno type	Time point	DIM+ co-loc w/repeat	DIM+ Not co-loc w/repeat	No DIM	# Cells	Total # Cells	% of cells w/DIM-repeat co-loc	% of cells w/DIM-repeat co-loc (Total)	adj. P value[2]
WT	20	50	24	31	105	323	47.62	41.49	1
		48	35	35	118		40.68		
		36	21	43	100		36.00		
mrc1AQ		35	20	37	92	221	38.04	37.56	
		13	18	8	39		33.33		
		35	34	21	90		38.89		
WT	30	55	23	31	109	330	50.46	43.03	<.0001
		46	16	56	118		38.98		
		41	12	50	103		39.81		
mrc1AQ		22	24	54	100	264	22.00	25.38	
		13	13	14	40		32.50		
		32	27	65	124		25.81		
WT	40	52	23	44	119	345	43.70	46.67	<.0001
		56	17	52	125		44.80		
		53	12	36	101		52.48		
mrc1AQ		15	10	65	90	244	16.67	16.80	
		9	8	24	41		21.95		
		17	17	79	113		15.04		
WT	50	42	24	52	118	334	35.59	39.52	<.0001
		50	17	49	116		43.10		
		40	14	46	100		40.00		
mrc1AQ		11	12	73	96	255	11.46	16.08	
		11	9	26	46		23.91		
		19	12	82	113		16.81		
WT	60	32	17	60	109	339	29.36	30.68	<.0001
		40	15	73	128		31.25		
		32	15	55	102		31.37		
mrc1AQ		11	11	72	94	248	11.70	13.31	
		7	7	34	48		14.58		
		15	9	82	106		14.15		

[2]Bonferroni-adjusted Chi Sq.

Table S10: Primers used in this study

Primer sequence	Source	Identifier
5’-CCTCAGCCTGGCCGAAAGAAAGAAA-3’	Eton Bioscience Inc.	NewCAGfor
5’-CAGTCACGACGTTGTAAAACGACGG-3’	Eton Bioscience Inc.	NewCAGrev
5’-GGTCAGGGCCTCAGCCTGGCCGAAA-3’	Eton Bioscience Inc.	2Step_pAG32_F
5’-GGCCGGCGGAACGGGGCTCGAA-3’	Eton Bioscience Inc.	2Step_pAG32_R
5'-TAATACGACTCACTATAGGG-3’	Eton Bioscience Inc.	T7-20
5'-CCCAGGCCTCCAGTTTGC-3’	Eton Bioscience Inc.	CTG rev2
5'-AACTACTGGGAAAACATTCG gttttagagctagaaatagcaagttaaaataagg-3'	Eton Bioscience Inc.	Rad53_pRCC_F
5'-CGAATGTTTTCCCAGTAGTT tccgatcatttatctttcactgcggag-3'	Eton Bioscience Inc.	Rad53_pRCC_R
5'-TTGCCACAGTAAAGAAAGCCATTGAAA GAACTACTGGGAAAACATTCGCCGTGGCGATTA TAAGTAAACGCAAAGTAATAGGCAATATGGAT GGTGTGAC-3'	Thermoscientific	Rad53^{K227A} Repair Template_F
5'-GTCACACCATCCATATTGCCTATTACTT TGCGTTTACTTATAATCGCCACGGCGAATGTTTT CCCAGTAGTTCTTTCAATGGCTTTCTTTACTGTG GCAA-3'	Thermoscientific	Rad53^{K227A} Repair Template_R
5'-TAGACAAGAATCGTTGCTTG gttttagagctagaaatagcaagttaaaataagg-3'	Eton Bioscience Inc.	Cep3-pRCC-F
5'-CAAGCAACGATTCTTGTCTA tccgatcatttatctttcactgcgg-3'	Eton Bioscience Inc.	Cep3-pRCC-R
5'-CGGGTCTTTGGTACCGTTGAATAAGC TTAGACAAGAAGCGTTGCTTGAAGAAGAGGAC GAAAACAATACGGAACCAAGTGACTTCAGAAC TATTGTAGAA-3'	Thermoscientific	Cep3^{S575A} Repair Template_F
5'-TTCTACAATAGTTCTGAAGTCACTTGGT TCCGTATTGTTTTCGTCCTCTTCTTCAAGCAACG CTTCTTGTCTAAGCTTATTCAACGGTACCAAAGA CCCG-3'	Thermoscientific	Cep3^{S575A} Repair Template_R
5'-CGGGTCTTTGGTACCGTTGAATAAGC TTAGACAAGAAGAGTTGCTTGAAGAAGAGGAC GAAAACAATACGGAACCAAGTGACTTCAGAAC TATTGTAGAA-3'	Thermoscientific	Cep3^{S575E} Repair Template_F

5'-TTCTACAATAGTTCTGAAGTCACTTGGTTCCGTATTGTTTTCGTCCTCTTCTTCAAGCAACTCTTCTTGTCTAAGCTTATTCAACGGTACCAAAGACCCG-3'	Thermoscientific	Cep3^{S575E} Repair Template_R
5'-GTATTGAAAACCACTTCAAAGGGGCCCAATAGCACATTTATAGAATTCAAGTCaaaaaaaaaaaaaaaGACGGAAGTCCA-3'	Eton Bioscience Inc	mad2Δ Repair Template
5'- GGGCAGAAAGGTAACGCTTG gttttagagctagaaatagcaagttaaaataagg-3'	Eton Bioscience Inc	Mad2-pRCC-F
5'-CAACGTTACCTTTCTGCCC tccgatcatttatctttcactgcgg-3'	Eton Bioscience Inc	Mad2-pRCC-R
5'CCTGTTGGAATAACGCGAAAAGAAGGGTACGGCGTAATCAGACTCACTATAGGGAGACCG-3'	Eton Bioscience Inc	pAG32_ChrIV+909k_F
5'-GTCTATGACTTAGTTCTCCGAAAAAAATACAAGCCGTGTGCAGCTGAAGCTTCCCAGGCC-3'	Eton Bioscience Inc	pAG32_ChrIV+911k_R
AAAAAAATACAAGCCGTGTGGTTTTAGAGCTAGAAATAGCAAGTTAAAATAAG	Eton Bioscience Inc	chriv-inc_CRISPR_F
CACACGGCTTGTATTTTTTTGATCATTTATCTTTCACTGCGGAG	Eton Bioscience Inc	chriv-inc_CRISPR_R

Supplemental References

[S1] A. J. Osborn and S. J. Elledge, “Mrc1 is a replication fork component whose phosphorylation in response to DNA replication stress activates Rad53,” *Genes Dev.*, vol. 17, no. 14, pp. 1755–1767, Jul. 2003, doi: 10.1101/gad.1098303.

[S2] X. A. Su, V. Dion, S. M. Gasser, and C. H. Freudenreich, “Regulation of recombination at yeast nuclear pores controls repair and triplet repeat stability,” *Genes Dev.*, vol. 29, no. 10, pp. 1006–1017, May 2015, doi: 10.1101/gad.256404.114.

[S3] C. Horigome *et al.*, “PolySUMOylation by Siz2 and Mms21 triggers relocation of DNA breaks to nuclear pores through the Slx5/Slx8 STUbL,” *Genes Dev.*, vol. 30, no. 8, pp. 931–945, Apr. 2016, doi: 10.1101/gad.277665.116.

[S4] P. Heun, T. Laroche, M. K. Raghuraman, and S. M. Gasser, “The Positioning and Dynamics of Origins of Replication in the Budding Yeast Nucleus,” *J. Cell Biol.*, vol. 152, no. 2, pp. 385–400, Jan. 2001.
